# The role of immune checkpoints in modulating cancer stem cells anti-tumor immune responses: implications and perspectives in cancer therapy

**DOI:** 10.1186/s13046-025-03514-4

**Published:** 2025-11-13

**Authors:** Ola J. Hussein, Menatallah Rayan, Tasnim R. Matarid, Dana Elkhalifa, Hanan H. Abunada, Lubna Therachiyil, Ashraf Khalil, Shahab Uddin, Cristina Maccalli, Hesham M. Korashy

**Affiliations:** 1 Department of Pharmaceutical Sciences, College of Pharmacy, QU Health, Qatar University, Doha, Qatar; 2 Department of Pharmacy, Aspetar Orthopedic and Sports Medicine Hospital, Doha, Qatar; 3 College of Health and Life Sciences, Hamad bin Khalifa University, Doha, Qatar; 4 Biomedical Research Center, QU Health, Qatar University, Doha, Qatar; 5 Translational Research Institute, Academic Health system, Hamad Medical Corporation, Doha, Qatar; 6 College of Pharmacy, Dubai Medical University, Dubai, United Arab Emirates; 7 Dermatology Institute Academic Health System, Hamad Medical Corporation, Doha, Qatar; 8 Research Branch, Sidra Medicine, Doha, Qatar; 9 Unit of Biotherapy, IRCCS Ospedale Ploclinico San Martino, Genova, Italy

**Keywords:** Cancer stem cells, Stemness, Cancer immunotherapy, Immune checkpoints, Immune evasion, Therapy resistance, Immune checkpoint inhibitors

## Abstract

Cancer stem cells (CSCs) are a minor subpopulation of tumor cells characterized by self-renewal capacity and stemness features and are responsible for tumor progression and therapy resistance. Several studies have shown that CSCs possess immunomodulatory properties that allow them to evade from immune responses. One of the mechanisms by which CSCs can escape from immune cells recognition and killing is represented by the overexpression of immune checkpoints (ICPs). The observation that cancer patients may still display or acquire resistance to immunotherapy despite targeting the PD-1/PD-L1 axis, highlights the importance of other ICPs as potential mediators of immune resistance. In this review, we summarize the immunomodulatory properties of CSCs and comprehensively discuss the crosstalk between these cells and selected ICPs (i.e., B7-H3, B7-H4, CD200 and CD155, VISTA, TIGIT, CD47, CD70, CEACAMs, and galectins) that are thought to be involved in CSC mediated immune evasion. Open questions regarding the immunological profile of CSCs, especially in relation to ICPs expression and their underlying regulatory mechanisms, are also addressed. Improved immunological profiling of CSCs will contribute to the identification of prognostic and predictive biomarkers for cancer patients and the development of effective therapeutic interventions that may lead to the eradication of malignant tumors.

## Introduction

The achievements over the last decade in immunotherapy have changed the paradigm of cancer therapy [1]. Notably, the clinical development of monoclonal antibodies (mAbs) antagonizing the signaling of inhibitory molecules, known as immune checkpoint (ICP) inhibitors (ICIs), such as programmed cell death 1/ligand 1 (PD-1/PD-L1) and cytotoxic T-lymphocyte-associated protein 4 (CTLA-4) inhibitors, represent a major breakthrough in immunotherapy [1]. These agents have shown unprecedented success in achieving long-lasting durable responses in a number of aggressive solid tumors [2, 3]. However, a significant proportion of cancer patients failed to respond to these therapies due to primary or acquired resistance despite expressing PD-L1 [1, 4, 5].

Tumors encompass multiple heterogeneous subpopulations that differ in their genetic, phenotypic and functional characteristics and, as a result, their response to cancer therapies. Among these subpopulations, the presence of a minor subpopulation known as cancer stem cells (CSCs) or cancer-initiating cells (CICs) endowed with self-renewal and multipotency characteristics was shown to play a crucial role in mediating cancer recurrence and therapy resistance [5, 6]. Recently, there has been a growing interest in studying the immunomodulatory properties of CSCs, including their effects on response to ICIs. In this review, we summarize the CSCs' hallmarks and their immunological characteristics. Then, we comprehensively review the available evidence pertaining to the crosstalk between CSCs/CICs and various ICPs (i.e., PD-1/PD-L1, CTLA-4, B7-H3, B7-H4, CD200 and CD155, VISTA, TIGIT, CD47, CD70, CEACAMs, and galectins) and their role in the impairment of cancer immunosurveillance. Lastly, we discuss potential immunotherapies and their combination to overcome CSCs/CICs-mediated immunosuppression.

## Hallmarks of CSCs/CICs

One of the recent theories for cancer recurrence and therapy resistance is the presence of a stem-like cell population inside the tumor, known as cancer stem cells (CSCs) or cancer-initiating cells (CICs). CSCs/CICs represent a minor subpopulation of tumor cells that are resistant to standard therapies and endowed with stemness properties, mainly self-renewal capacity, infinite proliferation and multipotency [5, 7]. These cells are thought to be responsible for tumor initiation, progression, metastasis, recurrence and resistance to conventional therapies, ultimately contributing to treatment failure [8]. The ability of these cells to cycle from proliferation to quiescence, overexpression of anti-apoptotic and drug efflux proteins, and upregulation of DNA repair molecules are among the principal molecular alterations that play a role in driving their resistance to therapy [9, 10]. This underscores the pivotal role of CSCs in carcinogenesis, highlighting them as crucial targets for anti-cancer therapies [11].

Although CSCs were primarily thought to be rare and quiescent cell types modulating a unidirectional hierarchy within the tumors, further research revealed their unique phenotypic plasticity and self-renewal capabilities, allowing them to adapt within the tumor microenvironment (TME), which contributes to genetic heterogeneity and intratumoral heterogeneity in cancers [12–14]. CSCs were primarily discovered in leukemia as characterized by the CD34^+^/CD38^−^ phenotype by John and Bonnet in 1997 [15] and were subsequently identified in solid tumors such as colon, breast, ovarian, pancreatic, hepatocellular carcinoma and thyroid cancer [16].

CSC emergence has been primarily hypothesized to be either from differentiated normal or cancer cells, stem or progenitor cells, or through cell fusion of cancer cells with stem cells or cancer cells with differentiated cells. Moreover, hypoxia, cytokines as well as elevated nitric oxide (NO) concentration in the TME play a crucial role in the development and maintenance of CSCs [17]. The TME is essential for the functioning of CSCs that could be owing to the interactions of these cells with other components and factors within the TME, collectively referred to as the CSC niche. This niche protects the CSCs from anti-tumor immune responses and modulates their migratory properties. The CSC niche includes cancer cells, the extracellular matrix (ECM), endothelial cells, fibroblastic cells, immune cells, perivascular cells or their progenitor cells, cytokines and growth factors [9, 15, 18].

CSCs are characterized by the increased expression of certain stemness markers that are exploited for their identification. Based on their subcellular localization, they are broadly classified into intracellular and cell-surface markers. The cell surface markers that are identified in both solid and hematological malignancies include CXCR4, LGR5, EpCAM, ProC-R, LINGO2, CD24, CD44, CD117, CD110, CD133, CD166, CD87, CD90, CD29, CD61, CD70, CD371 and CD49f. The intracellular markers involve cytoplasmic proteins and transcription factors including SOX-2, NANOG, OCT3/4, Krüppel-like factor 4 (KLF4), aldehyde dehydrogenase (ALDH), Letm1, SALL4, BMI1, Musashi-1/2, AFP and Dcamkl-1 [17, 19].

The isolation of CSCs can be accomplished using a variety of methods, each of which has both advantages and drawbacks (Table 1). It was initially performed based on density and size differences using density-gradient centrifugation [13]. However, this technique employed Percoll, a colloidal silica coated with polyvinylpyrrolidone (PVP), which had a limitation of media toxicity, raising concerns about its safety [20]. Due to advances in cell sorting-techniques, major sorting techniques employed now include fluorescence-activated cell sorting (FACS), magnetic-activated cell sorting (MACS), and side population (SP) sorting, microfluidics, colony/sphere formation-based isolation, and sequencing-based sorting [21].

**Table 1:** Advantages and drawbacks of different CSC isolation methods

Sorting Method	Principle	Advantages	Drawbacks	Ref
Density-gradient Centrifugation	Differences in size and density	• Simple and cost-effective	• Separation medium toxicity	[13, 20, 21]
Magnetic-activated Cell Sorting (MACS)	Immunomagnetic bead sorting	• Sterile sorting • Large-scale sorting • Minimal impact on cell viability	• High cost • Complex Operation • Only measures cell surface markers • Measures a single target at a time • No universal validated marker for CSC sorting	[22–24]
Fluorescence-activated Cell Sorting (FACS)	Fluorescently labeled antibodies	• Assess metabolic activity • Assess intracellular and cell surface markers • Can screen multiple targets simultaneously	• Difficult to maintain sterility • Large number of cells required • Significant impact on cell viability • No universal validated marker for CSC sorting	[24, 25]
Side population (SP) Sorting	Efflux of Hoechst 33342 dye	• Easy to conduct	• Dye toxicity • Inadequate resolution between SP cells and non-SP cells	[26–29]
Colony/Sphere Formation	Ability of CSCs to form colonies and spheres in culture	• Label-free method	• Laborious • Low Yield • May isolate non-CSCs	[30–32]
Microfluidics-based Sorting	Physical properties of CSCs	• Label free • Rapid • Reproducible	• May isolate non-CSCs	[33]
RNA Sequencing	Transcriptional differences between CSCs and Non-CSCs	• Comprehensive CSC characterization	• Overlooking signals from critical populations	[34]

Both FACS and MACS identify CSCs using cell surface biomarkers such as CD24, CD44, CD133, EpCAM, and others [35]. MACS is an affinity-based technique that utilizes antibody-labeled magnetic beads to target cell surface antigens, allowing bound cells to be retained in a column while unbound cells get washed away [22, 23]. MACS enables sterile sorting and is suitable for large-scale sorting. Furthermore, MACS beads exert minimal mechanical pressure on cells, and the components of the beads (iron oxides and polysaccharide coating) are non-toxic, leading to minimal impacts on cell viability and biological behavior. However, it is time-consuming, difficult to operate, costly, cannot detect intracellular targets, and is difficult to multiplex [24]. On the other hand, FACS uses fluorescent antibodies to label cells thereby sorting the CSCs from other cell types [24]. Unlike MACS, FACS can assess metabolic activities, intracellular targets and can screen several markers simultaneously [25]. Nevertheless, FACS can be stressful to cells, which affects viability and biological behavior. Furthermore, given the rarity of the CSC population, a large number of cells is required to obtain a good yield of CSCs, leading to high experimental costs. Finally, if the sorted cells are to be maintained in culture for downstream applications, strict sterile conditions of the FACS facility are required, which are difficult and expensive to maintain [24]. In general, a limitation of using biomarkers to isolate CSCs is that there is uncertainty regarding how well each marker defines the CSC population, as CSCs can vary or change their gene expression during different tumor stages and different culture conditions [25]. SP sorting exploits the enhanced drug-efflux abilities of CSCs which is mainly attributed to the elevated expression of a transporter protein such as ABCG2. In this assay, CSCs efflux the Hoechst 33342 dye, leading them to appear as a dim tail in the cell population [26–28]. Although SP sorting is a relatively simpler method to isolate CSCs, it is limited by dye cytotoxicity and inadequate resolution between SP cells and non-SP cells [29].

Label-free methods can also be utilized to sort and isolate CSCs. The ability of CSCs to form colonies and spheres in culture can also be used to isolate CSCs. The cells can be seeded in low-density serum-free conditions in the presence of epithelial growth factor (EGF) and basic fibroblast growth factor (bFGF). Under such conditions, CSCs will form colonies or spheres, while non-CSCs will die, allowing the isolation of CSCs. However, this process is laborious, has low yield, and may isolate contaminating non-CSCs [30–32]. Additionally, a novel microfluidic technology was recently developed to sort CSCs based on size, elasticity, and adhesiveness, allowing rapid, label-free, reproducible, and efficient CSC enrichment. Nevertheless, non-CSCs may be accidentally isolated if they share similar physical properties to CSCs [33].

Other methods of CSCs isolation includes the integration of high-throughput sequencing with cell sorting to isolate CSCs based on their gene expression profiles, enabling more comprehensive characterization of CSCs rather than relying on a single marker or property [34]. Nevertheless, traditional bulk sequencing is limited by the possibility of overlooking signals from small critical cell populations such as CSCs. To address these limitations, single-cell RNA sequencing and spatial transcriptomics are now being increasingly employed to allow a more sophisticated understanding of CSCs and intra- and inter-tumor heterogeneity [36, 37].

Reports suggest that CSCs use specific cellular signaling pathways for their growth and self-renewal such as Wnt/β-catenin [38] Sonic hedgehog (Shh) [39], Notch [40] and BMI1 [41]. Moreover, many signaling pathways involved in cellular growth and proliferation in normal cells such as JAK/STAT, PI3K/AKT, mTOR, NF-κB [31, 42] are often dysregulated in CSCs. CSCs exhibit active states of both proliferation and quiescence. In the quiescent state, metabolic activity is significantly reduced, making them resistant to therapeutic interventions. This can be chiefly attributed to the fact that chemotherapeutic drugs mainly target actively dividing cells and miss quiescent cells that are usually in the G1 or S phase of the cell cycle [43]. Furthermore, CSCs can efflux chemotherapeutic drugs, evade immune responses, scavenge reactive oxygen species (ROS), induce angiogenesis, undergo metabolic reprogramming, and possess enhanced DNA repair mechanisms [44].

Another key hallmark of CSCs/CICs that has attracted much attention is their ability to evade immune surveillance and mediate resistance to immunotherapies [5, 6, 45]. Studies suggested that CSCs can mediate immune evasion through the release of immunosuppressive proinflammatory factors (e.g., IL-10, IL-13, IL-4, Galectin-3, and TGFβ) [8, 9]. These factors drive the differentiation of immune cells towards suppressive subtypes, such as T regulatory cells (Tregs) and myeloid-derived suppressor cells (MDSCs) [46]. Moreover, CSCs express low levels of human leukocyte antigen (HLA) molecules and tumor-associated antigens, which renders these cells invisible to immune surveillance, leading to the inability of cytotoxic immune cells to recognize and kill CSCs/CICs [6, 47]. On the other hand, several studies revealed that CSCs/CICs express elevated levels of inhibitory ICP molecules, such as PD-1/PD-L1, CTLA-4 and B7-H3 [48–50]. The engagement of ICPs with immune cells, mainly T cells, contributes to inefficient immune responses and promotes CSCs survival [2]. Nevertheless, the mechanisms behind the immunomodulating characteristics of CSCs are still largely unknown. Therefore, a comprehensive characterization of the genomic, epigenetic and immunological profile of CSCs will aid in gaining a better understanding of the regulatory pathways.

## Cancer-associated ICP molecules and their interplay with stemness features

**Fig. 1:**
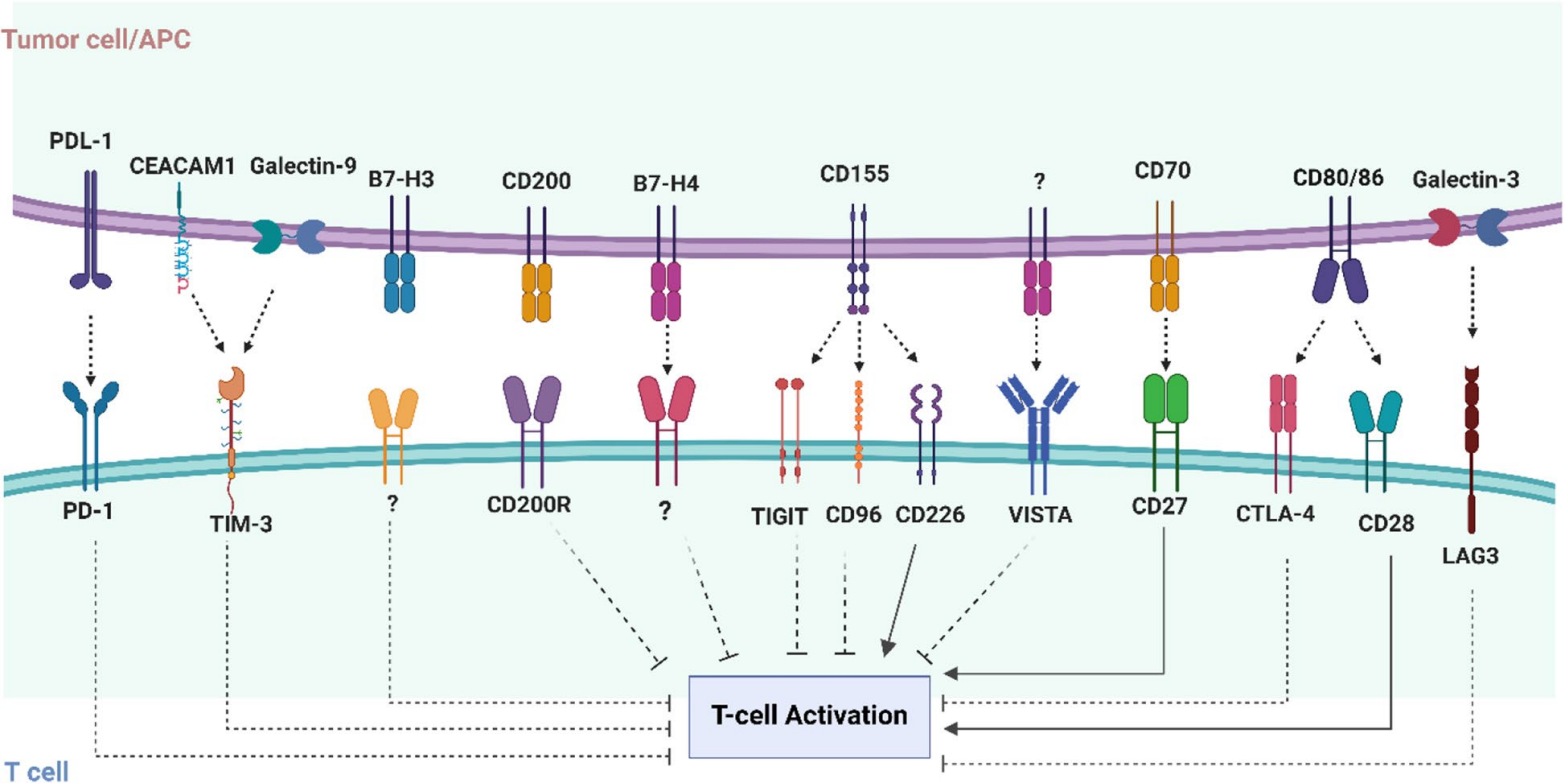
Tumor-associated immune checkpoints (ICPs) and their effects on mediating T cell activation. This figure illustrates the complex interplay between tumor-associated ICPs and T cell activation in the tumor microenvironment (TME). It shows how inhibitory ICPs (e.g., PD-1/PD-L1, CTLA-4, B7-H3, B7-H4, CD155, CD200, TIGIT, and VISTA) suppress T cell activation, thereby enabling tumor cells to evade immune detection and destruction. Conversely, stimulatory checkpoints including CD27, CD28 and CD226, are also shown to highlight the balance between immune activation and suppression. The figure highlights the dual role of ICPs in regulating immune homeostasis and promoting immune evasion in cancer

ICPs are critical molecules found on cell surfaces that regulate immune functions, restraining autoimmunity and unregulated immune responses through intercellular communication [51, 52]. ICPs function as gatekeepers in ligand-receptor pairs, regulating the interaction between antigen-presenting cells (APCs) and both innate and adaptive immune system cells, especially T cells [53]. ICPs are categorized as inhibitory or stimulatory checkpoints according to their effects on immune responses (Fig. 1). Inhibitory ICPs are frequently overexpressed on tumor cells or within their microenvironment, thereby effectively dampening immune responses [53]. While the principal function of ICP molecules is to mediate immune responses, it has been discovered that ICP molecules expressed on cancer cells also contribute to enhancing various malignant characteristics, including self-renewal, resistance to therapies, apoptosis evasion, angiogenesis and metastasis [48, 54–57].

**Fig. 2:**
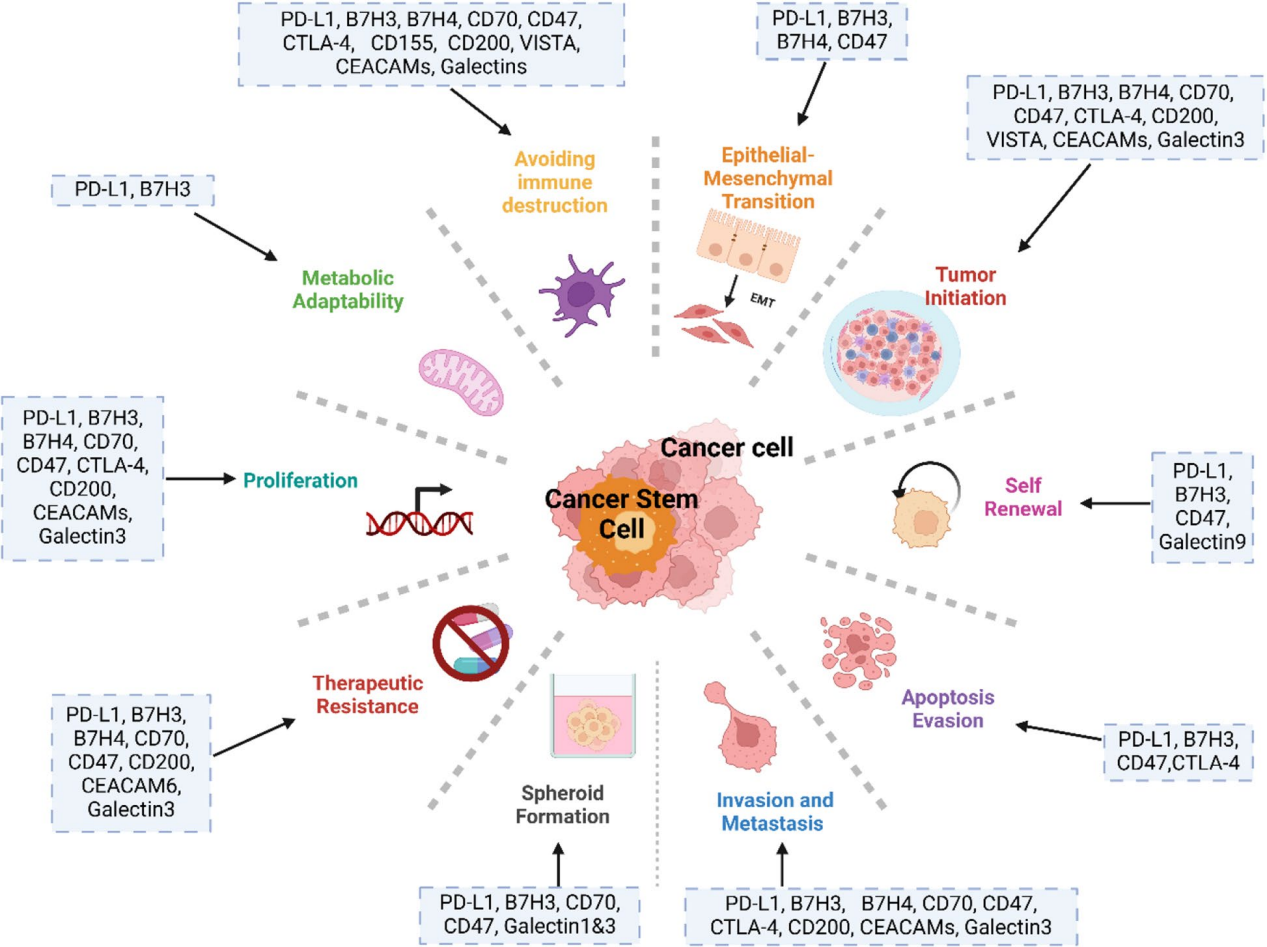
Key hallmarks of cancer stem cells (CSCs) and their crosstalk with immune checkpoints (ICPs). This figure summarizes the defining hallmarks of CSCs including self-renewal, therapy resistance, metabolic adaptability, invasion, metastasis, and immune evasion. Tumor-associated ICPs that regulate each of these hallmarks are highlighted, emphasizing the complex crosstalk between CSCs and the immune system

**Fig. 3:**
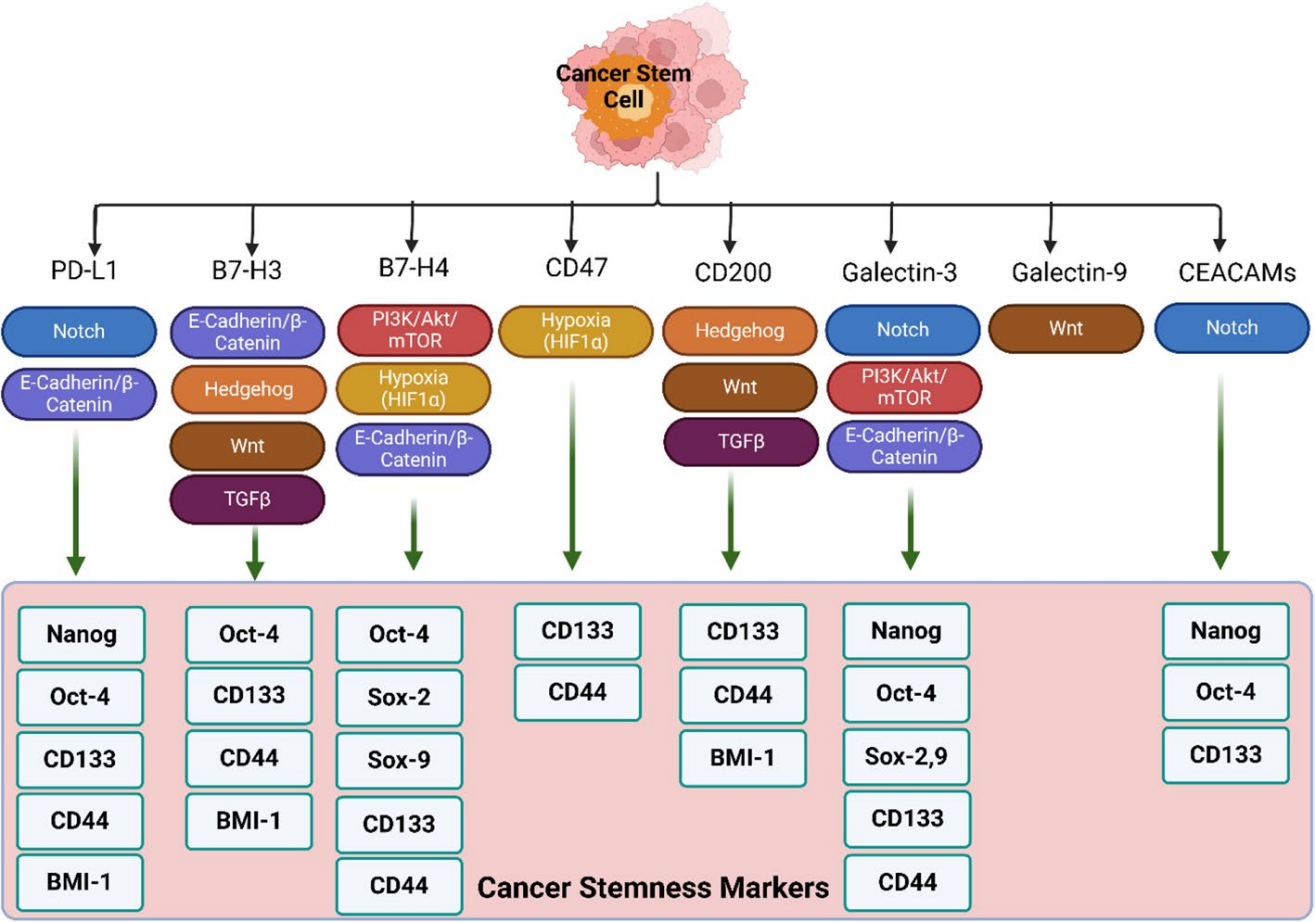
Impact of tumor-associated immune checkpoints on stemness-related signaling pathways and the expression of stemness markers. This figure demonstrates the mechanistic link between immune checkpoint (ICP) signaling and the maintenance of cancer stem cell (CSC) characteristics. It shows how the engagement of inhibitory ICPs (e.g., PD-1/PD-L1, CTLA-4, B7-H3, B7-H4, CD155) triggers downstream signaling pathways such as PI3K/AKT, Wnt/β-catenin, JAK/STAT, and NF-κB, which promote CSC survival, self-renewal, and therapy resistance. The figure also depicts how ICP signaling influences the expression of key stemness markers, including CD44, CD133, SOX-2, and OCT-4, thereby enhancing CSC plasticity and adaptability in the tumor microenvironment

To date, two major inhibitory ICP signaling pathways have been heavily studied, namely PD-1/PD-L1 and CTLA-4. Blocking of these ICPs can release the breaks in the immune system and enhance the anti-tumor immune response [3, 53]. In the past few years, targeting these inhibitory ICPs by mAbs has emerged as an important pillar in cancer therapy. Particularly, anti-PD-1 (e.g., nivolumab) and anti-CTLA-4 (e.g., ipilimumab) therapies have shown remarkable success in achieving long-lasting durable responses in several types of tumors [3, 53]. However, a substantial portion of cancer patients do not respond to these therapies, possibly due to variations in the characteristics of their TME [53, 58]. Remarkably, recent studies suggested that the presence of CSCs/CICs may play a role in protecting cancer cells from immune destruction through differential expression of ICPs, alteration of their downstream signaling or through promoting immunosuppressive tumor microenvironment [50, 52]. Thus, the primary resistance to ICIs or its associated recurrence may be at least partially mediated through the failure of these therapies to eliminate CSCs/CICs. While several studies reported that CSCs from different types of cancers express a high level of PD-L1, the full array of ICP receptors expressed by CSCs or their underlying regulatory mechanisms has not been fully investigated [52, 59, 60]. Available studies have been heavily skewed towards the PD-L1/PD-1 axis, with limited data on the expression and regulation of other ICPs (e.g., PD-L2, CD200, B7-H3, B7-H4, CD155, LAG-3, CD47, CD70, CD80, CD86, CEACAM-1, HVEM). Noting that cancer patients may still possess resistance to anti-PD-L1 therapy despite expressing PD-L1 highlights the importance of other ICPs that can mediate immune evasion in distinct or complementary pathways, especially in the highly resistant CSC subpopulation [61–63]. The following section summarizes the available knowledge on tumor-associated ICPs (i.e., PD-L1/PD-1, CTLA-4, B7-H3, B7-H4, CD155, CD200, VISTA, TIGIT, CD47, CD70-CD27, CEACAMs and galectins) that have been shown to be involved in mediating CSC immune evasion, with a focus on recently discovered ICPs (Table 2). These studies highlighted the crucial role of ICPs in regulating key hallmarks of CSCs, including tumor initiation, self-renewal, apoptosis evasion, invasion and metastasis, epithelial-mesenchymal transition (EMT), spheroid formation, therapeutic resistance, proliferation, metabolic adaptability, and immune evasion (Fig. 2). Mechanistically, the same ICPs have been found to play a critical role in regulating stemness-related signaling pathways, thereby influencing the expression of stemness markers (Fig. 3).

**Table 2:** Tumor-associated ICPs detected in CSCs and their underlying cellular mechanisms

**Molecule**	**Type of cancer**	**Study design**	**CSCs enrichment** **method**	**Expression in CSCs/CICs vs. differentiated tumor cells**	**Activity in CSC/CICs**	**Ref**
**CTLA-4** **(CD152)**	GBM	In vitro (Primary CSC lines and differentiated tumors)	Selective media and hypoxic conditions	↑		[64]
	Cutaneous melanoma	Bioinformatics analysis (TCGA)	Stem cell-related genes	–	High-risk group is more sensitive to CTLA-4 immunotherapy	[65]
	RCC	In vitro (786-O cell line) In vivo xenograft mouse model	Sphere formation	Both CSCs and non-CSCs express low levels (∼0.8%)		[66]
	Bladder carcinoma	Bioinformatics analysis	-	Indirect correlation	FGF-2 induces EMT, cell proliferation and CSC self-renewal FGF-2-expressing tumors tend to express high levels of CTLA-4, PD-1 and PD-L1	[67]
	RCC	Bioinformatics analysis (TCGA, ICGC)	Stem cell-related genes	–	Tumors with the highest CSC concentration had the highest sensitivity to anti-CTLA-4	[68]
	HCC	Bioinformatics analysis of the TCGA HCC cohort	mRNAsi risk score*	↑		[69]
	Breast cancer	Human tissues	Stem cell marker (Sca-1, also known as Ly6E/K)	Increased expression of Ly6E/K correlated with ↑ CTLA-4	Increased expression of Ly6E/K correlated with increased expression of the ICP molecules PD-L1 and CTLA-4	[70]
	NSCLC	In vitro (cell lines) Human tissues	CD166^+^	↑ expression in CSCs than non-CSCs and in tumor than non-tumor adjacent tissues	c-MET/CTLA-4 bispecific antibodies specifically target lung CSCs and inhibit hepatocyte growth factor-mediated tumor development, including proliferation, migration, and apoptosis	[71]
	Melanoma	Indirect Evidence Bioinformatics analysis of the TCGA melanoma cohort	Stem cell markers (CD20, CD38, ABCB5, CD44, etc.)	Indirect inverse correlation (↓ CTLA-4, PD-1 and PD-L1) in group with high stemness markers)		[72]
	Adrenocortical carcinoma	Bioinformatics Analysis (TCGA)	mRNAsi risk score*	↓ CTLA-4 in high-mRNAsi group	Lower expression of PD-L1, CTLA-4, and TIGIT was observed in the high-mRNAsi group	[73]
**PD-L1** **(B7-H1)**	PCa	Immunostaining and quantitative analysis using samples from PCa patients	CSC- related markers (CD44^+^/CD133^+^)	↑ in CSCs (CD44 +/CD133 +) vs. non-CSCs	PD-L1 expression is positively correlated to an increased proportion of CSCs and poor prognosis	[74]
	Breast cancer	In vitro (MCF-7 and BT-549)	Spheroid formation	↑ in tumor spheres vs. differentiated cells	PD-L1 expression in CSCs is regulated by epigenetic modifications and contributes to immune evasion	[75]
	Breast cancer	In vitro (MDA-MB-231 Hs578T, BT-549, and SUM-149)	Spheroid formation	↑ in up to 3-fold on breast CSC-like cells vs. differentiated-like cancer cells	PD-L1 overexpression on CSCs maintains the stemness through the NOTCH3/mTOR pathway	[76]
	EC	In vivo xenograft mouse model	CSCs-related markers (ALDH1, CD133^+^)	↑ in ECSCs vs. non-stem-like cancer cells	PD-L1 expression influences CSC self-renewal and proliferation, regulated by hypoxia HIF-1α and HIF-2α activation	[77]
	CRC	In vitro (HT-29) In vivo xenograft NOD/SCID mouse model	CSCs related markers (CD133^+^)	↑ CD133^+^ CSCs compared to CD133^−^ cells	B7-H1 enables immune evasion via co-expressing EMT markers; involved in tumor invasiveness and metastasis	[78]
	CRC	In vitro (HT29 and HCT116) In vivo xenograft nude mouse model	Spheroid formation CSCs-related markers (CD133^+^/CD44^+^)	↑ in CD133^+^ CD44^+^ CSCs vs. non-CSCs	The expression of PD-L1 enhances CSC self-renewal and expansion through HMGA1 pathways. In turn, it contributes to chemoresistance and tumorigenicity	[79]
**PD-1**	SCLC	In Vitro (primary SCLC cells and established cell lines) In vivo xenograft CD1-nude mouse model Co-cultures (SCLC cells & PBMCs)	CSCs-related markers (CD90^+^/CD44^+^)	↑ in CD44 ^+^ CD90 ^+^ CSCs vs. non-CSCs	PD-1 and its ligands promote CSC immune evasion in addition to resistance to cytotoxic T lymphocytes (CTLs)	[50]
	HNSCC	In vitro (cell lines)	CSCs-related markers (BMI1^+^)	↑ in BMI1^+^ CSCs enriched after combination treatment with cisplatin + anti-PD-1	Inhibition of BMI1 and PD-1 blockade eradicate CSCs, activates anti-tumor immunity, and prevents cancer metastasis and relapse	[80]
**B7-H3 (CD276)**	HCC	Bioinformatic analysis of HCC cohort from TCGA	mRNAsi risk score*	↑	-	[69]
	CRC	In vitro cell lines (SW480 and Caco-2 cells) In vivo xenograft mouse IHC of CRC tissues	CSCs-related markers (CD133, CD44 and OCT4)	NA	Promote EMT and cancer stemness by ↓ E-cadherin and ↑ N-cadherin, Vimentin, CD133, CD44, and OCT-4	[54]
	CRC	CRC tissue samples	CSCs-related markers (CD133^+^)	↑ in CD133^+^ CSCs	Co-expression with CD133^+^ was associated with higher tumor invasion, metastasis and poorer survival	[48]
	Ovarian cancer	In vitro (eight ovarian cancer cell lines including platinum- and taxane-resistant cell lines)	ALDH^bright^	NS	Blocking B7-H3 with a mAb reduces the number of CSCs	[81]
	Glioma	In vitro (isolated patients’ primary cancer cells)	Spheroid formation	NS	↑ cell invasion and sphere formation	[82]
	GBM	In vitro (cell lines)	Spheroid formation	↑ CSCs spheroids	-	
	Cervical cancer	In vitro (HeLa cell line) In vivo xenograft mouse model	Spheroid formation	NA	↑ sphere formation, proliferation, self-renewal, tumor growth, and chemotherapy resistance	[55]
	HNSCC	In vitro (cell lines) In vivo patient-derived xenograft (PDX) HNSCC tissue samples	CSCs-related markers (BMI1^+^)	↑ in BMI1^+^ CSCs	Aids in immune evasion during cancer initiation, development and metastasis Blockade of B7-H3 eliminates CSCs and reduces tumor growth and metastasis	[83]
	Breast cancer	In vitro (cell lines) In vivo xenograft mouse model	CSCs-related markers (CD24^low ^CD44^high^)	↑	↑ CSC population and cancer progression through MVP/MEK signaling	[84]
	Breast cancer	Bioinformatics analysis of the TCGA breast cancer cohort	Basal/stem gene signature	Correlated with basal/stem and invasiveness gene signatures	Associated with stemness-related pathways (Wnt, TGF-β, and Hedgehog signaling)	[85]
	PCa	In vitro (cell lines)	ALDH^+ ^CD44^+^	↑ in CSCs	Blocking of B7-H3 by CAR T cells induced higher cytotoxicity in CSCs as compared to bulk of tumor	[86]
**B7-H4 (VTCN1)**	GBM	In vitro (cell lines) GBM tissues and CSF	CSCs-related markers (CD133^+^)	↑ in CD133^+^ GBM CSCs	Mediate the immunosuppression interplay between CD133^+^ GBM and tumor-infiltrating macrophages/macroglia	[87]
	RCC	In vitro (cell lines)	Spheroid formation	Weakly positive for B7-H4	-	[66]
	Brain tumors	In vitro (isolated patients’ primary cancer cells) In vivo xenograft mouse model	Spheroid formation CSCs-related markers (CD133^+^)	Expressed in the non-dividing (quiescent) fraction of CSCs	-	[88]
	NSCLC	In vitro NSCLC tissues	Spheroid formation Stemness markers	Correlated with the expression of cancer stemness-related proteins	Silencing of B7-H4 reduced cancer cells invasion and migration through activating AMPK/mTOR signaling, which was associated with lower expression of stemness-related markers (SOX-2, SOX-9, CD44) and EMT markers (Snail, vimentin), while the expression of E-cadherin was upregulated Colocalized with (SOX-2 and SOX-9) in the nucleus	[89]
	ESCC	In vitro (cell lines) ESCC tissues	-	↑ in less differentiated ESCC	Associated with poor prognosis and less CD8^+^T cell infiltration Correlated with the expression of cyclin D1, p27, and PI3K/Akt/NF-κB signaling	[56]
	Leukemia	In vitro (cell lines) In vivo xenograft mouse model	CSCs-related markers (CD34^+^)	Expressed on a fraction of CICs	Promote differentiation of CICs and inhibit tumorigenesis through PTEN/AKT/HIF1α/RCOR2/RUNX1 pathways	[90]
**CD200** **(OX-2)**	Multiple cancers	In vitro (cell lines) Gene expression	Stem cell markers (cancer-type specific)	↑ in CSCs population	CD200 co-expressed with CSC markers in breast (CD44^+^CD24^−^), prostate (CD44^+^), colon (CD133) and brain cancers, suggesting a potential involvement of CSCs in immune evasion	[91]
	CRC	In vitro (multiple cell lines)	CSCs-related markers (CD133^+^)	Expressed on a subset of CD133^+^ cells of one cell line and absent in the remaining		[92]
	HNSCC (HPV-positive and HPV-negative)	In vitro (cell lines) In vivo xenograft mouse model	-	Correlated with stemness markers	Promoted chemoresistance and correlated with the expression of cellular proliferation and stemness-related markers (BMI1 and Shh), both of which were upregulated with CD200 overexpression	[57]
	CRC	In vitro (cell lines) Microarray analysis	CSCs-related markers (CD44^+^CD133^+^)	↑ in CD44^+ ^CD133^+^ CSCs	CD200^+^ cells possessed higher colony formation ability, invasiveness, and enriched CSCs population (CD44^+^CD133^+^) CD200^+^ possessed several differentially expressed genes that are involved in cell proliferation, metastasis, and immune response Share gene expression similarity with CD44^+^CD133^+^ CSCs, suggesting its potential utility as a CSCs biomarker	[93]
	AML	Blood and bone marrow patients’ samples In vivo xenograft mouse model	Functional validation by xeno-transplantation CSCs-related markers (CD45^dim^)	↑ CD200 in Leukemia stem cells	CD200^+^ cells have higher tumor formation ability in vivo CD200^+^ cells capture both CD34^+^ and CD34^−^ populations of leukemia stem cells in AML patients	[94]
	Breast cancer	Bioinformatics analysis of the TCGA breast cancer cohort	Basal/stem gene signature	Correlated with basal/stem and invasiveness gene signatures	Associated with stemness-related pathways (Wnt, TGF-β, and Hedgehog signaling) May act as CSC-specific ICP to mediate immune evasion Significantly co-expression with CD47	[85]
**VISTA**	Breast cancer	Human tissue microarray	-	-	VISTA expression associated with poor differentiation	[95]
	Pancreatic cancer	Human tissues	-	-	VISTA expression is reduced with higher differentiation	[96]
	Melanoma	In vitro (cell lines); In vivo xenograft mice model; Human tissues	FOXD3	-	VISTA expression promotes tumor onset and alters the immune microenvironment FOXD3 represses VISTA expression	[97]
**CD155**	GBM	In vitro	Spheroid	↑ in gCSCs as compared to serum-cultured cells	-	[52]
	Breast cancer	Mouse-derived mammary carcinoma cell line	CD44^+ ^CD24^−/low^	Comparable levels to non-CSCs	Expression increased upon cell irradiation	[98]
**CD47 (B6H12)**	AML	Human AML samples In vivo (NOG mouse)	Lineage^−^CD34^+^CD38^−^CD90^−^	⟷ CD47 in CSC fraction as in bulk leukemia cells ↑ CD47 in CSCs as compared to normal stem cells	Increased expression of CD47 correlated with worse overall survival Blocking CD47 with mAbs enabled phagocytosis of leukemia stem cells and inhibited their engraftment in vivo	[99]
	CRC	CRC tissues	CD44	Correlated with expression of CD44	Promotes malignancy of CRC and enhances stemness of cancer cells CD47 along with CD44 are potentially involved in mediating resistance to PD-1/PD-L1 inhibitors	[100]
	CRC	In vitro (macrophages and tumor cells) In vivo xenograft mouse model Human normal and tumor tissues	Lineage^− ^ESA^+ ^CD44^+^	-	Blocking CD47 with human mAbs induced the phagocytosis of colorectal CSCs by macrophages	[101]
	HCC	In vitro (cell lines) HCC tissues	Spheroid formation	↑ in CSCs as compared to differentiated counterparts	Promotes tumor initiation, self-renewal and metastasis Knockdown of CD47 inhibited stem cell characteristics CD47 regulates stemness by inducing cathepsin S/protease-activated receptor 2 signaling	[102]
	Lung cancer	Lymph node aspirates from lung cancer patients	CSCs defined by panel of surface markers as CD45^−^/CXCR4^+^/EpCAM^+^/CD133^+^/CD44^+^/CD90^+^	↑ in CSCs as compared to mature tumor cells	-	[103]
	Lung cancer	In vitro (cell lines) In vivo mouse xenograft model Lung tumor and normal tissues	CD133^+^	↑ in CSCs as compared to tumor cells and normal stem cells	The expression of CD47 correlated with poor survival Blocking CD47 with mAb enabled macrophage phagocytosis of CSCs and inhibited tumor growth in vivo	[104]
	Bladder cancer	Human tumor tissues In vitro In vivo mouse xenograft model	CD44^+^	↑ in CSCs as compared to rest of the tumor	Blocking CD47 by mAbs induced macrophage engulfment of bladder cancer cells	[105]
	PDAC	In vitro (primary pancreatic cancer cells) In vivo mouse xenograft model Human tissues	Sphere formation In vivo limiting dilution tumorigenicity assay Sorting (CD47^+^CD133^+^)	↑ in CSCs than the rest of the tumor and normal nonmalignant cells	Targeting CD47 enhanced phagocytosis of primary pancreatic CSCs and induced their apoptosis	[106]
	TNBC	In vitro (cell lines) Bioinformatics analysis (TCGA data)	Sphere formation	-	Treating CSCs with CD47 antibody decreased proliferation and asymmetric cell division by downregulating EGFR and KLF4 in a SIRPα-independent mechanism	[107]
	Breast cancer	In vitro (cell lines) Bioinformatics analysis (TCGA data)	Sphere formation	↑	CD47 deficiency induces CSCs depletion Transcription of CD47 is activated by HIF-1 under hypoxic conditions, which is known to promote CSCs	[108]
	Ovarian cancer	In vitro (cell lines)	Sorting (ALDH^high^)	↑ 2-fold higher in CSCs as compared to non-CSCs	-	[109]
	Osteosarcoma	In vitro	CD44^+^	80–90% of CSCs express CD47	-	[110]
	ESCC	In vitro (primary ESCC cells) IHC of ESCC tissues	Sphere formation Stemness marker (CD133)	Correlated with CD133 expression	High CD47/CD133 expression was associated with poor prognosis CD47^+^ cells formed larger spheres than CD47^−^ cells Inhibition of CD47 inhibited self-renewal and eliminated CSC pool	[111]
**CEACAM1L**	GBM	In vitro (mouse primary neural cells, human neural stem cells and GICs) In vivo xenograft mouse model Human and mouse brain tissues	Primary GICs cultured in CSC media	70% of GICs express CEACAM1L ↑ xpression in GICs compared to neural stem cells	↑ Colony formation and angiogenesis in GICs Activates c-Src/STAT3 signaling ↑ expression of stemness-related genes (NOTCH3 and SOX-1-4, 9)	[112]
**CEACAM 1,3,5,6**	Cervical cancer	In vitro (*CaSki cell line)* In vivo xenograft mouse model Primary human cervical SCC biopsy samples	Spheroid formation	↑ in CSCs spheroids	CEACAM^+^ cells showed higher invasion, colony formation, and tumor-forming abilities as well as upregulation of Notch signaling and expression of stemness-related genes (NANOG, OCT4, ABCC3)	[113]
**CEACAM5**	CRC	Circulatory tumor cells (CTCs) from CRC patients	-	CEA^+^/CK^+^/CD133^+^ CTCs	CEA/CK/CD133-positive CTC/CSCs showed a worse prognosis	[114]
	Pancreatic cancer	Bioinformatics analysis of pancreatic cancer cohort from TCGA and CTRP	mRNAsi risk score*	↑	↑ CEACAM5 expression associated with invasive cell-enriched signature and MSI^ +^ CICs enriched signature	[115]
**CEACAM6**	Breast cancer	In vitro (MCF-7 cell line) In vivo xenograft mouse model	Spheroid culture	-	CEACAM6 Ab-conjugated doxorubicin (DOX)-loaded liposomes (CDDOXL) selectively showed increased uptake and cell-killing effects in breast CSCs with high CEACAM6 expression levels	[116]
	CRC	In vitro (Caco2 cell line) In vivo xenograft mouse model CRC tissues	Primary spheroid culture	Association of CD66c^bright^ with stemness marker (CD133)	CD66c^bright^ showed higher tumorigenicity and was able to differentiate and reform CD66^−^-cells	[117]
**Galectin-1**	Breast cancer	In vitro (multiple cell lines)	Spheroid culture	↑ galectin-1 expression	↑ sphere formation ↑ CD44	[118]
**Galectin-3**	HCC	In vitro (BMOL and AML-12) Gene expression analysis of 221 HCC patients	-	Associated with higher CD44, SOX-9, EpCAM	BMOL cells cultured with media from non-tumorigenic AML-12 cells depleted from galectin-3 exhibited a significant decrease in stemness markers (CD44, SOX-9, EPCAM, CK19) ↑ expression correlated with poor survival in HCC	[119]
	USC	In vitro (ARK1, ARK2, SKOV3, HUVECs, normal fibroblasts) In vivo (ARK1 Gal-3-CTRL and KO xenograft mouse model) USC human tissues	-	-	Inhibition of Gal-3 decreases spheroid formation and tumorigenesis	[120]
	Ovarian cancer	In vitro (multiple cell lines) In vivo (xenograft mouse model) Ovarian cancer human tissues	-	Correlated with the expression of stemness-related genes	Inhibition of Gal-3 reduces sphere formation, proliferation, drug resistance, invasion and migration and the expression of stemness-related genes (NANOG, OCT-4, SOX-2) as well as NOTCH1 and its target genes (Hey1 and Hes1)	[121]
	Lung cancer	In vitro (multiple cell lines)	Sphere formation	-	Gal-3 activates EGFR/c-Myc/SOX-2 pathway and enhances sphere formation OCT4 enhances Galecitn-3 expression	[122]
	TNBC	In vitro (MDA-MB-231, MCF-7) In vivo (xenograft mouse model) Breast cancer human tissues	CD44^+^CD24^−^	↑ Gal-3 associated with CD44^+^CD24^−^	Gal-3 promotes cancer stemness, tumor progression, aggressiveness and drug resistance in TNBC in vitro and in vivo Blocking Gal-3 reduces stemness markers (OCT4, NANOG and SOX-2)	[123]
	RCC	In vitro (Caki‐1, ACHN, A‐498) In vivo (xenograft mouse model) Human RCC tissues	Spheroid culture	↑ in CSCs	Inhibiting Gal-3 reduced invasion, colony formation, sphere formation, drug resistance and expression of stemness‐related genes (NANOG and SOX-2) by regulating CXCR2	[124]
	Lung cancer	In vitro (multiple cell lines) In vivo (xenograft mouse model) Human lung cancer tissues	Spheroid culture	↑ in CSCs	Inhibition of Gal-3 downregulated stemness-related genes, sphere formation, drug resistance, and tumorigenicity whereas its overexpression promoted stemness Gal-3 expression correlated with CD133 and β-catenin expression which were associated with higher tumor progression	[125]
	HER2^+^ breast cancer	In vitro (SKBR3 and JIMT1) In vivo (xenograft mouse model) Bioinformatics analysis	-	-	↑ Gal-3 was associated with poor prognosis Gal-3 promoted malignancy by activating PI3K/Akt and stemness by targeting NOTCH1 signaling, resulting in enhanced trastuzumab resistance	[126]
	Breast cancer	In vitro (GI-101A and GI-LM2) In vivo (xenograft mouse model) Human breast cancer tissues	Spheroid culture	↓ Gal-3 in CSCs	↓ Gal-3 expression correlates with poor outcomes in node-positive breast cancer Loss of Gal-3 enhanced sphere formation, ALDEFLUOR activity, and drug resistance and in vivo tumorigenicity On the other hand, lower Gal-3 was associated with lower canonical Wnt signaling and Lgr5	[127]
**Galectin-8**	GBM	In vitro (primary CSCs and differentiated tumor counterparts) In vivo (xenograft mouse model) GBM human tissues	Primary CSCs	↑ Galectin-8 expression	Gal-8 enhances autophagy and promotes stemness though activating mTORC1-TFEB axis ↑ Gal-8 correlated with poorer prognosis and higher stemness markers (CD133, SOX-2, and OLIG2)	[128]
**Galectin-9**	AML	Bone marrow (BM) and peripheral blood samples of AML patients In vivo (NSG xenograft mouse model)	CD34^+^CD38^−^ enrichment	-	↑ β-catenin and promotes canonical Wnt to stimulate self-renewal and propagation of LSCs, independent of Wnt ligands	[129]
	Acute lymphoblastic leukemia (ETP-ALL)	In vitro (multiple cell lines) In vivo (patient-derived xenograft mouse model) Primary samples (banked blood or bone marrow samples from patients with relapsed/refractory ETP-ALL, all with activating NOTCH1 mutations)	-	-	Stem-like cells could differentiate into mature leukemia cells with high expression of galectin-9, resulting in immune suppression and accumulation of dysfunctional CD8+ T cells	[130]
**CD70**	PDAC	Proteomic profiling of fresh frozen cells IHC analysis of PDAC tissues	CD24^+^	↑ in CD24^+^ as compared to CD24^−^ cells	Expression and colocalization with CD24 suggest a potential role in the progression of PDAC and a potential therapeutic target	[131]
	CML	In vivo (xenograft mouse model)	CD34^+^	-	CD70 is induced in LSCs by TKI where it mediates drug resistance through activating Wnt signalling Combination of TKIs with CD70 blockade synergistically reduced cell proliferation and colony formation in vitro and eliminated CD34^+^ LSCs in xenograft model	[132]
	Breast cancer	In vitro (multiple cell lines) 122 primary breast cancer samples	-	-	CD70^+^ but not CD70^−^ was associated with self-renewal, differentiation potential, and higher tumor sphere formation ability and lung metastasis	[133]
	GBM	In vitro In vivo (xenograft mouse model)	-	-	Silencing of CD70 downregulated stem-related markers (CD44 and SOX-2), inhibited tumor growth and migration, promoted immune suppression by attracting monocyte-derived M2 macrophage CD70-specific CAR T cell therapy showed a profound anti-tumor effect without observed toxicity	[134]
	AML	In vitro In vivo (xenograft mouse model) Patients' blood samples	CD45^dim ^SSC^low ^CD90^+ ^CD34^+^	Expressed in AML stem/progenitor cells but not on hematopoietic stem/progenitor cells from healthy donors (Expressed in CD34^+^ leukemia stem cells)	Activate stem cells related genes including Wnt pathway, and enhance cell proliferation Blocking CD70/CD27 axis induced differentiation in AML blasts and stem progenitor cells, reduced cell growth and colony formation and improved survival of AML xenograft mice	[135]
	AML	In vivo (xenograft mouse model) Phase I/II clinical trial	Lin^− ^CD90^− ^CD34^+ ^CD38^−^	↑ in LSCs	Blocking CD70/CD27 with cusatuzumab, a human αCD70 mAb eliminated LSC in vitro and in vivo Cusatuzumab monotherapy and in combination with azacytidine in previously untreated older AML patients, resulted in reducing LSCs and induced stemness gene signature related to myeloid differentiation and apoptosis potentially leading to deep and durable remission	[136]
	GBM	In vitro (patient-derived GBM) In vivo (xenograft mouse model) Multi-omics	Spheroid formation	↑ in CSC-enriched GBM as compared to GBM cohort in TCGA	Essential for sphere formation and proliferation Knockdown reduces tumorgenicity	[137]

^*^ mRNA expression-based stemness index (mRNAsi), an index developed by machine learning to quantitatively identify cancer stemness features

↑ Over-expressed; ↓ Downregulated; ABCB5: ATP-binding cassette sub-family B member 5; ALDH: Aldehyde dehydrogenase; AML: Acute myeloid leukemia; AMPK: Adenosine monophosphate-activated protein kinase; B7-H3: B7 homolog 3; B7-H4: B7 homolog 4; CD: Cluster of Differentiation; CD47: Integrin-associated protein; CD70: Tumor necrosis factor ligand; CD155: Poliovirus receptor; CD200: OX-2 membrane glycoprotein; CEACAMs: Carcinoembryonic antigen-related cell adhesion molecules; CICs: Cancer-initiating cells; CRC: Colorectal cancer; CSCs: Cancer stem cells; CSF: Cerebrospinal fluid; CTLA-4: Cytotoxic T-lymphocyte-associated antigen 4; EGFR: Epidermal growth factor receptor; EMT: Epithelial-mesenchymal transition; ESCC: Esophageal squamous cell carcinoma; FGF-2: Fibroblast growth factor 2; FOXD3: Forkhead box D3; Galectins: Beta-galactoside-binding proteins; GBM: Glioblastoma; GICs: Gastric initiating cells; HCC: Hepatocellular carcinoma; HER2: Human epidermal growth factor receptor 2; HNSCC: Head and neck squamous cell carcinoma; HPV: Human papillomavirus; ICGC: International Cancer Genome Consortium; ICPs: Immune checkpoint proteins; IL: Interleukin; JAK/STAT: Janus kinase/signal transducers and activators of transcription; Ly6E/K: Lymphocyte antigen 6 complex, locus E/K; LSCs: Leukemia stem cells; mAb: Monoclonal antibody; mRNAsi: mRNA stemness index; mTOR: Mammalian target of rapamycin signaling; MMPs: Matrix metalloproteinases; NA: Not assessed; NF-κB: Nuclear factor kappa-light-chain-enhancer of activated B cells; NS: Non-significant; NSCLC: Non-small cell lung cancer; PD-1: Programmed cell death protein 1; PD-L1: Programmed death-ligand 1; PDX: Patient-derived xenograft; PI3K/AKT: Phosphoinositide 3-kinase/Protein kinase B pathway; RCC: Human renal carcinoma; Sca-1: Stem cell antigen-1; SHP-1/2: Src homology 2 containing protein tyrosine phosphatases; TCGA: The Cancer Genome Atlas; TIGIT: T cell immunoreceptor with Ig and ITIM domains; VISTA: V-domain immunoglobulin suppressor of T cell activation

### CTLA-4

CTLA-4 (CD152) was the first discovered ICP and is regarded as a principal inhibitory regulator of the activation of T cells [138]. CTLA-4 is a component of the immunoglobulin superfamily and is present in Tregs and activated cytotoxic T cells [139, 140]. CTLA-4 exhibits considerable homology with the CD28 receptor, sharing identical ligands, specifically B7.1 (CD80) and B7.2 (CD86), which are expressed on APCs [141]. Although both CD28 and CTLA-4 can bind to the same ligands, they exert contrasting effects on T cell functionality. In this context, CD28 delivers a costimulatory signal essential for the activation and survival of T cells, whereas CTLA-4 inhibits T cell activation, proliferation, and differentiation via numerous pathways [142–144]. CTLA-4 promotes immune evasion primarily by competitively binding to CD80/CD86 proteins with a 10- to 100-fold higher affinity than CD28, thereby suppressing T cell activation [145, 146]. Furthermore, CTLA-4 has been documented to attenuate T cell responses by trans-endocytosis and the breakdown of B7 proteins from APCs surfaces, as well as disrupting T cell receptor (TCR) function [147]. Besides cell-extrinsic processes, CTLA-4 has been demonstrated to function via cell-intrinsic pathways by recruiting phosphatases, leading to the dephosphorylation of crucial signaling molecules downstream of TCR and CD28 [142, 148, 149]. Alteration of CTLA-4 expression has been associated with various autoimmune disorders, including type 1 diabetes [150] and rheumatoid arthritis [151]. In cancer, increased CTLA-4 expression in the TME is crucial for immune evasion. Of note, elevated levels of CTLA-4 in nasopharyngeal [152], breast [153, 154], thymus [155] and esophageal [156] malignancies have been associated with unfavorable patient outcomes. Consequently, immunotherapies targeting CTLA-4 have been formulated and have demonstrated significant potential in augmenting anti-tumor immune responses. These drugs have created a novel paradigm for the treatment of cancer patients, including those in advanced stages. For example, ipilimumab, a mAb targeting CTLA-4, was the first ICP inhibitor to receive approval upon demonstrating enhanced overall survival in metastatic melanoma patients [157, 158]. Ipilimumab is currently utilized as a monotherapy for melanoma and in conjunction with anti-PD-1 (nivolumab) for other malignancies, including renal, colorectal and lung cancer [159, 160]. Nonetheless, the clinical effectiveness of anti-CTLA-4 therapy is constrained and confined to a certain group of cancer patients. Consequently, it is essential to elucidate the processes governing CTLA-4-mediated immune evasion and to find indicators of response to enhance the efficiency of these therapies.

While CTLA-4 has been primarily studied for its role in regulating anti-tumor immune responses, emerging studies have begun to shed light on its potential role in CSCs. To date, available data on the expression of CTLA-4 in CSC subpopulations is still limited and is an area of active research. Di Tomaso et al. demonstrated that both primary CSCs derived from glioblastoma (GBM) patients and their differentiated tumor counterparts express CTLA-4, with CSCs exhibiting higher expression levels [64]. Similarly, Li et al. revealed that CTLA-4 is overexpressed in CD166^+^ lung CSCs as compared to the non-CSC population isolated from primary non-small-cell lung cancer (NSCLC) tumor tissues or cell lines [69]. In the same study, treating lung CSCs with a bispecific antibody targeting CTLA-4 and c-MET effectively inhibited cell proliferation and migration and reduced tumor volume in vivo*.* These effects were associated with the inhibition of Treg cells and upregulation of effector T cells [69]. Besides, AlHossiny et al. showed that increased expression of the stem cell marker (Ly6E/K) in breast cancer cells correlated with higher expression of CTLA-4 [70]. Additionally, analysis of the hepatocellular carcinoma (HCC) cohort from The Cancer Genome Atlas (TCGA) database showed that high mRNA expression-based stemness index (mRNAsi) risk score, an important index of CSCs, was associated with higher expression of CTLA-4 [69]. Consistently, Wang et al. reported that renal cell carcinoma (RCC) tumors with high expression levels of stem cell genes had the highest sensitivity to immunotherapy targeting CTLA-4 [68]. On the other hand, analyzing melanoma and adrenocortical carcinoma cohorts with the same risk score, mRNAsi showed an inverse correlation with CTLA-4 expression [72, 73]. Notably, melanoma patients with low-risk mRNAsi were found to respond better to anti-CTLA-4 therapy [65]. Mechanistically, blocking CTLA-4 was shown to suppress key CSC features, such as ALDH activity, sphere formation, and in vivo tumorigenesis [161]. These observations highlight the importance of studying CTLA-4 checkpoint and its downstream signaling, especially in the CSCs subpopulation.

### PD-1/PD-L1

PD-1 and its ligand PD-L1 are a well-characterized ICPs that are broadly used in clinical cancer immunotherapy due to their vital role in suppressing immune responses and facilitating self-tolerance by decreasing T cell inflammatory activity. PD-1 (CD279) belongs to the type I transmembrane glycoprotein superfamily and is a member of the CD28 family [162]. PD-1 is a coinhibitory receptor widely expressed on the surface of activated T cells, B cells, monocytes, neutrophils, natural killer (NK) cells, myeloid cells, and dendritic cells (DCs) [162–165]. PD-L1 (B7-H1 or CD274) and PD-L2 (B7-DC or CD273) are members of the B7 family and transmembrane ligands for PD-1 with PD-L1 being the most dominant regulator [162–164]. PD-L1 is expressed in a wide range of cells, including immune cells like T cells, B cells, mast cells, DCs, and normal tissue cells, whereas PD-L2 is expressed primarily in DCs, mast cells, macrophages, and some B cells [162].

The interaction between PD-1 and its ligands, PD-L1 or PD-L2, with the subsequent suppression of immune responses is crucial for preventing autoimmune diseases; however, it can be manipulated by cancer cells to escape the immune system [166, 167]. Mechanistically, binding of PD-1 to PD-L1 or PD-L2 on activated T cells, leads to tyrosine phosphorylation of the cytoplasmic domain and triggers recruitment of a Src homology 2 containing protein tyrosine phosphatase 1 and 2 (SHP-1/2) to the signaling motifs on the PD-1 cytoplasmic tail, the immunoreceptor tyrosine-based switch motif (ITSM). SHP-1 and SHP-2 dephosphorylate key proteins in the TCR signaling pathway, thus suppressing downstream signaling pathways, involving PI3K, Akt, MAPK, RAS, and ERK, and reducing interleukin-2 (IL-2) secretion [162, 168]. This downregulation in the immune system arrests the T cell cycle, reduces the anti-apoptotic proteins such as Bcl-xl, and inhibits the production of IL-2 and interferon gamma (INF-γ). Consequently, this leads to cancer cell survival and proliferation by evading the host immune detection [169]. This mechanism has been recognized as the main mechanism of adaptive immune resistance that enables tumors to be resistant to the immune response and thus is considered as a promising therapeutic target [170, 171]. In this context, several mAbs targeting PD-1 and PD-L1 ICPs, including nivolumab, pembrolizumab, avelumab, and durvalumab, have been approved by the FDA as clinical immunotherapies for various types of cancer, such as metastatic melanoma, NSCLC and metastatic Merkel cell carcinoma (MCC) [163].

Several studies have discussed the expression of the ICPs, PD-1 and PD-L1, in different types of CSCs. Study findings revealed that a high level of PD-L1 was positively associated with low CD8^+^ T cell infiltration combined with an increase in pancreatic CD44^+^/CD133^+^CSCs [74]. Research data have also demonstrated that the expression of PD-L1 is associated with EMT and plays a role in human breast CSCs. PD-L1 was upregulated in both MCF-7 and BT-549 tumor spheres compared with the bulk of tumor cells, due to epigenetic modification in the PD-L1 promoter region [75]. In the same context, Mansour et al. reported that the activation of the NOTCH3/mTOR signaling pathway is responsible for the overexpression of PD-L1 on breast CSCs [76]. In this study, the PD-L1 level was upregulated by threefold in breast CSCs compared with more differentiated cancer cells, and the expression was associated with EpCAM^+^/CD44^high^/CD24^low ^alongside EpCAM^+^/CD90^+^CSCs [76]. Furthermore, a study on endometrial CSCs demonstrated that PD-L1 expression is elevated in the CSCs due to the activation of the hypoxia-inducible factors HIF-1α and HIF-2α, which are associated with CSCs' self-renewal and tumorigenicity [77]. In addition, the study showed that the knockdown of PD-L1 is correlated with impaired CSC proliferation, reduced expression of related genes, such as ALDH1, OCT-4, NANOG, CD133, SOX-2, and a lower number of CD133^+ ^ECSCs [77]. Another study reported that CD133^+ ^HT29 cells presented EMT phenotypes and high B7-H1 expression, suggesting that CSCs might express B7-H1 to avoid immune responses [78]. A further study reported upregulation in PD-L1 levels in CD133^+^CD44^+ ^colorectal (CRC) cells and even higher in CD133^+ ^CD44^+ ^colorectal CSCs and CSC-enriched tumor spheres [79]. An in vitro experimental study using small cell lung cancer (SCLC) stem cells found that the interaction between T cells and CD44^+^/CD90^+ ^CSCs resulted in an upregulation in ICPs, such as PD-1, CTLA-4, lymphocyte-activation gene 3 (LAG-3), and T cell immunoglobulin and mucin-domain containing-3 (TIM-3) [50]. A study by Jia et al. reported that the polycomb complex protein (BMI1^+^) CSCs were enriched following treatment with a combination of cisplatin and anti-PD-1. Moreover, the inhibition of BMI1 effectively eliminates CSCs and strengthens the efficacy of PD-1 blockade therapy due to their synergistic effect. In turn, it eliminates CSCs, inhibits the metastasis of head and neck squamous cell carcinoma (HNSCC), and prevents relapse [80]. A recent study by Tout et al. found that miRNA-15a downregulates PD-L1 expression in colorectal cancer (CRC) stem cells, indicating that miRNA modulation could be integrated with existing therapies to improve CSC elimination [172]. Collectively, these studies highlight the role of the PD-1/PD-L1 axis and underscore the importance of targeting this pathway to improve future cancer treatment outcomes.

### B7-H3 (CD276)

B7-H3 (CD276) is a member of the B7 protein superfamily, which is essential in modulating the responses of activated T cells [6, 54]. B7-H3 was first recognized as an ICP present on the surface of APCs or macrophages [6, 173]. B7-H3 is present at low levels in normal tissues but exhibits markedly elevated expression across a wide array of malignancies [2, 48]. A soluble version of B7-H3 was found at elevated levels in the serum of NSCLC patients compared to healthy controls, which was correlated with a greater tumor burden [82]. The soluble form is believed to be released due to the cleavage of the B7-H3 cell surface receptor from tumor and immune cells by matrix metalloproteinases (MMPs). The ligand for B7-H3 remains unidentified; nevertheless, it is hypothesized to be expressed on activated T cells, as evidenced by its inhibitory impact on T cell-driven anti-tumor immunity [2, 82]. B7-H3 has recently been identified to have non-immunological pro-tumorigenic functions, including promoting invasion, metastasis, anti-apoptotic activity, thereby potentiating therapy resistance, and stemness, the latter being less well-established [2, 173]. Although substantial evidence supports the tumor-promoting role of B7-H3, no FDA-approved drugs targeting this molecule are currently available [174]. The absence of a known receptor for B7-H3 presents a challenge for pharmacological targeting [174]. Identifying its receptor could significantly accelerate the development of effective B7-H3-targeted therapies. Nevertheless, due to its critical role in cancer progression, various strategies such as mAbs and chimeric antigen receptor (CAR) T-cell therapy, have been explored as potential therapeutic approaches in clinical trials [174].

Several reports revealed that B7-H3 is selectively enriched in CSCs. For instance, B7-H3 was elevated in CD133^+^ colorectal CSCs, GBM spheroids, and BMI1^+^ HNSCC stem cells [48, 83]. On the contrary, fewer reports showed that B7-H3 is equally elevated in differentiated cancer cells as well as CSCs, particularly glioma-initiating cells and ALDH^bright^ ovarian CSCs [81]. Functional studies suggested that B7-H3 potentiates EMT and cancer stemness by reducing E-cadherin and inducing N-cadherin vimentin, CD133, CD44 and OCT-4 [54]. Thus, overexpression of B7-H3 was accompanied by enhanced sphere formation, proliferation, self-renewal, cell invasion and metastasis [48, 55, 82, 83]. Mechanistically, Liu et al. demonstrated that B7-H3 promotes stemness through its interaction with major vault protein (MVP), which activates MAPK signaling [84]. Silencing MVP impairs B7-H3–mediated MEK activation and significantly suppresses B7-H3-induced CSCs [84]. Consistently, analysis of breast cancer-specific data set from TCGA showed that B7-H3 was associated with stemness-related pathways, such as Wnt, TGF- β, and Hedgehog signaling [85]. Recent investigations by Wang et al. have revealed that CSCs utilize B7-H3 ICP to evade immune surveillance during HNSCC initiation, development and metastasis [83]. Remarkably, B7-H3 blockade led to CSCs elimination and inhibited tumor growth and metastasis through promoting anti-tumor immunity, particularly CD8^+^ T cell-dependent cytotoxicity [83]. Based on these findings, B7-H3 was suggested as a functional cell surface marker to isolate CSCs from HNSCC [83]. Yet, further preclinical and clinical studies are still needed to confirm the role of B7-H3 as a biomarker for the identification of CSCs and to explore it as a target alone or in combination to overcome therapeutic resistance.

### B7-H4 (VTCN1)

B7-H4 is a type I transmembrane protein belonging to the B7 family that is encoded by the VTCN1 gene [2, 56, 88]. B7-H4 is prominently expressed in numerous malignant cells and tumor-associated macrophages, where its expression is linked to tumor aggressiveness and decreased survival rates [2, 56, 87–89]. The receptor for B7-H4 remains unidentified; however, it seems to be expressed on activated T-lymphocytes [2]. Binding of B7-H4 to its receptor provides inhibitory signals to T cells, thus hindering the activation, proliferation, and cytokine generation of CD4^+^ and CD8^+^ T cells [87, 88]. Furthermore, B7-H4 plays a role in the proliferation of MDSCs [175]. Similar to B7-H3, soluble B7-H4 is present in the bloodstream of cancer patients, and elevated levels have been correlated with a poorer prognosis [87]. Furthermore, the overexpression of B7-H4 markedly enhanced the anti-apoptotic effects, tumorigenesis, and invasion, indicating that other tumor-specific capabilities beyond its role in immune suppression, such as regulation of JAK2-STAT3 signaling and EMT [87]. Conversely, the knockdown of B7-H4 suppressed tumor growth, migration, invasion and colony formation, while enhancing apoptosis and inducing cell cycle arrest at the G0/G1 phase [176].

The potential role of B7-H4 in promoting CSCs/CICs has recently been investigated in a few studies. Yao et al. revealed that B7-H4 is selectively upregulated in CD133^+^ GBM stem cells as compared to non-CSCs [87]. Mechanistically, co-interaction of CD133^+^ CSCs and macrophages induces the expression of B7-H4 via IL-6 and IL-10, leading to immunosuppressive activity. Similarly, the expression of B7-H4 correlated with stemness-related markers (i.e., CD44, SOX-2, SOX-9, OCT4) in NSCLC and esophageal squamous cell carcinoma (ESCC) [57, 89]. Nevertheless, other studies showed that B7-H4 is expressed only in a subset of CD34^+^ leukemia stem cells and CD133^+^ brain CSCs/CICs, specifically the non-dividing (quiescent) fraction [88, 90]. Silencing of B7-H4 in vitro downregulated the expression of stemness-related proteins (i.e., SOX-2, SOX-9, CD44) and EMT markers (Snail, vimentin), resulting in lower migration and invasion, which supports its involvement in CSC-mediated tumorigenicity [89]. Whether targeting B7-H4 in vivo will lead to the elimination of CSCs/CICs and, subsequently, cancer eradication remains uncertain and thus, more studies are required to unleash the potential utility of B7-H4 as a biomarker for CSCs/CICs or tumor aggressiveness.

### CD200 (OX-2)

CD200 (OX-2) is a type I membrane glycoprotein that belongs to the highly conserved immunoglobulin family [57, 91, 93]. CD200 is normally expressed on neurons, endothelial cells, epithelial cells, thymocytes and a subset of T and B lymphocytes [57]. Notably, CD200 was found to be upregulated in several types of tumor cells as compared to their normal tissue counterparts [57, 93, 177]. The binding of CD200 on tumor cells with its receptor, CD200R, expressed on macrophages, DCs and T lymphocytes, delivers negative signals that promote immune tolerance [57, 178]. This interaction triggers an immunosuppressive signal through inhibition of T cell activation, switching of helper T cell phenotype from anti-tumor Th1 to immunosuppressive Th2, induction of Tregs and inhibition of macrophages [58, 178]. These findings suggest that CD200/CD200R1 pathway is exploited by cancer cells to induce immune tolerance and promote tumor progression [91, 93, 178]. Clinically, the expression of CD200 was associated with poor prognosis in multiple myeloma and acute myeloid leukemia [178]. Additionally, ectopic expression of CD200 in HNSCC was found to induce EMT and promote invasiveness and resistance to chemotherapy and radiotherapy [177, 178].

CD200 has been recently proposed as a potential new actionable CSC marker in various types of cancer. This is based on the observation of the association between CD200 expression and other CSC markers, such as CD44^+^/CD24^−^ in breast cancer, CD44^+^ in prostate cancer, CD133 in CRC, BMI1 and Shh in HNSCC [57, 92, 93]. This was further evidenced by the study of Ho et al. (2020), who functionally validated CD200 as a marker for leukemia stem cell activity using xenotransplantation assays [94]. In this study, CD200 was found to be selectively enriched in CD45^dim^ blasts as compared to the more differentiated CD45^high^ cells in acute myeloid leukemia (AML). Similarly, CD200 expression was upregulated in CD44^+^/CD133^+^ colorectal CSCs compared to CD44^−^/CD133^−^ colorectal non-CSCs, which was associated with higher colony formation, invasiveness, and CSC population [94]. However, Gemei et al. assessed the expression of CD200 in CSCs of five CRC cell lines and found that it is expressed only in a subset of CD133^+^ cells of the HT29 cell line, while it was completely absent in the other four cell lines (HCT116, Caco2, GEO and LS174T) [92]. Therefore, more studies on other types of tumors are required to assess if CD200 can be used as a universal biomarker for the detection of CSCs. Moreover, to the best of our knowledge, there are no studies that assessed the therapeutic potential of inhibiting CD200 as a mean to target CSCs, nor provided definitive evidence for its role in the induction of CSCs by knockdown or overexpression.

### CD155 (PVR, NECL5)

CD155, also known as poliovirus receptor (PVR) or NECL-5, is a type I transmembrane glycoprotein of the immunoglobulin superfamily [179–181]. CD155 has recently been identified as a potential target in cancer immunotherapy, where it is considered as another ICP found on tumor cells [182]. Although it is expressed at low levels in normal tissues, CD155 was found to be broadly overexpressed in several types of human malignancies, and was recognized as an unfavorable prognostic marker in multiple cancers [180–182]. CD155 contributes to immunosuppression in the TME through interaction with TIGIT on T cells or NK cells, resulting in inhibitory effects on their proliferation and cell-mediated cytotoxicity [180]. On the contrary, CD155 also binds to DNAM-1/CD226 on T cells and NK cells, which results in enhancing cytotoxic function [183]. Thus, the immunomodulatory role of CD155 is complex, and its function may be altered based on the surrounding environment. Despite this controversy, preclinical evidence indicates that CD155 blockade can augment the antitumor effects of PD-1–targeting ICIs, suggesting potential utility as an add-on therapy [179]. In addition to its role in modulating immune responses, CD155 also affects other cellular processes, such as cellular adhesion, proliferation, migration and differentiation. The role of CD155 in mediating cell invasion suggests that it might also be involved in the regulation of CSCs. Robilliard et al. (2019) found that CD155 is selectively upregulated in glioma spheroid culture of CSCs as compared to serum-cultured glioma cells [52]. On the other hand, Li et al. reported comparable levels of CD155 expression between CD44^+^/CD24^−/low^ breast CSC population and non-CSCs [98]. Therefore, the role of CD155 in CSCs remains largely unknown, and more studies are needed to assess its potential utility as a biomarker or therapeutic target.

### TIGIT

T cell immunoreceptor with immunoglobulin and ITIM domains (TIGIT) is a transmembrane glycoprotein that belongs to the immunoglobulin superfamily and is mostly expressed on activated and memory T cells, Tregs, and NK cells [184, 185]. It plays a crucial role in modulating immune responses and facilitating cancer immune evasion [183]. TIGIT interacts with two primary ligands, CD155 and CD112, which are expressed on APCs or tumor cells [73]. TIGIT has been identified as elevated in various malignancies, including lung cancer [186], melanoma [187], and gastric cancer [188]. Clinical studies indicate that elevated TIGIT expression correlates with unfavorable clinical outcomes across many malignancies [189]. At present, several antagonistic mAbs aimed at TIGIT are undergoing clinical trials as novel treatments for diverse tumor types [183]. Thus far, these drugs have not demonstrated significant anticancer efficacy as standalone treatments [184]. Nonetheless, the administration of anti-TIGIT mAbs in conjunction with other treatments, such as anti-PD-L1 or anti-TIM-3, has exhibited synergistic effects in many preclinical models [190]. TIGIT is a newly identified ICP that significantly contributes to tumor immune evasion, and its blockade by mAbs offers a promising treatment approach for cancer.

Despite its critical role in mediating immune evasion, no studies have directly examined the link between CSCs and TIGIT. Nevertheless, its ligand, CD155, has been shown to be upregulated in CSCs [52]. Besides, CD155 can be activated by the Shh signaling pathway, which plays a crucial role in self-renewal and cell fate determination in both normal cells and CSCs [191]. Given the therapeutic importance of this pathway, it is worth investigating the regulation of the TIGIT pathway in CSCs and whether its blockade could effectively target CSCs.

### VISTA

V-domain immunoglobulin suppressor of T cell activation (VISTA), also referred to as B7-H5, programmed death-1 homolog (PD-1H), SISP1, and DD1α, is a type I transmembrane protein that functions as an immunoregulatory checkpoint [192–194]. VISTA is expressed on hematopoietic cells, with the highest levels on myeloid cells, monocytes, and granulocytes, and to a lesser extent on T cells and NK cells, but not on B cells, which suggests a foundational role in maintaining basal immune tolerance and homeostasis [194, 195]. Furthermore, VISTA expression was observed at varied levels across different cancers [196, 197]. VISTA exhibits dual functionality as both a ligand and a receptor, with roles that vary depending on the cell type and tumor context [140, 193, 196–199]. Within the TME, VISTA functions predominantly as a negative immune checkpoint regulator, where it binds to T cells, inhibiting their activation, proliferation, and cytokine secretion, therefore facilitating immune evasion and tumor progression [194, 200–202]. Moreover, VISTA can act as a tumor-intrinsic suppressor under certain conditions. A recent study identified a conserved four-amino acid (NPGF) intracellular motif in VISTA that interacts with NUMB and sequesters it at endosomes, impairing epidermal growth factor receptor (EGFR) signaling and cellular proliferation in triple-negative breast cancer (TNBC) [203]. This suppressive effect is independent of immune cell interaction or canonical ligand binding and suggests a novel, immune-independent function of VISTA in constraining tumor growth [203].

From a therapeutic perspective, blockade of VISTA has been shown to elicit a robust induction of anti-tumor immunity and may enhance the efficacy of other immunotherapies [139, 198, 204]. The genetic deletion or antibody blockade of VISTA in both syngeneic and humanized AML mouse models significantly improved T cell–mediated immune clearance of AML cells, suggesting a role in both tumor cell–intrinsic and microenvironment-mediated immune suppression [195, 205]. Furthermore, a study by Moon et al. developed a dual-action lipid nanoparticle (dual-LNP) co-delivering VISTA-specific siRNA and a TLR9 agonist, effectively enhancing anti-tumor immunity in murine models. The dual-LNP outperformed monotherapies by promoting immune cell infiltration, DC activation, and cytotoxic T cell function. It presents a promising approach for treating immune-cold tumors through simultaneous VISTA silencing and TLR9 stimulation [206]. Therefore, VISTA is currently undergoing clinical assessments as a novel target for cancer immunotherapy [140, 207]. mAbs and small-molecule inhibitors targeting VISTA are undergoing phase 1 clinical trials (e.g., NCT02812875 and NCT02671955) and have demonstrated satisfactory tolerability and anti-tumor efficacy [192, 198]. The inhibition of VISTA notably augmented the activation of tumor-infiltrating lymphocytes and facilitated tumor-specific T cell responses, despite elevated PD-L1 expression [201]. Consequently, the PD-L1 and VISTA pathways are regarded as independent, and their concurrent dual blockade elicits synergistic anti-tumor responses, as evidenced by preclinical studies.

Despite its promising therapeutic potential, the role of VISTA as a prognostic biomarker remains complex and tumor type-specific. VISTA overexpression in certain malignancies, such as pancreatic, ovarian or lung cancer, correlates with improved survival rates, whereas its overexpression in other tumor types, such as melanoma, is associated with poor prognosis [208]. Therefore, the underlying regulators influencing the survival outcomes need to be further explored across different malignancies.

While direct evidence linking VISTA to CSCs is limited, some studies suggest a relationship between VISTA expression and cellular differentiation states. For instance, a study on human breast cancer revealed elevated VISTA expression in poorly differentiated cancer types [95]. Consistently, in pancreatic cancer, reduced VISTA levels were observed in tumors with more differentiated neoplasms [96]. Given that CSCs are frequently present in poorly differentiated tumors, VISTA may play a role in regulating stemness features and immune evasion in CSCs [209]. However, more studies are needed to validate these assumptions. On the other hand, the loss of VISTA expression has been linked to dedifferentiation into mesenchymal cells [210]. Besides, an inverse correlation between VISTA expression and EMT-associated gene expression was observed in malignant pleural mesothelioma tumors, suggesting that VISTA expression decreases during EMT [210]. Additionally, the stemness factor forkhead box D3 (FOXD3), previously identified as a mediator of melanoma therapy resistance, was shown to directly repress VISTA transcript and protein expression in melanoma cells [97]. These findings collectively indicate a potential relationship between VISTA and CSC biology. Further investigation into the mechanisms underlying VISTA's involvement in these developmental processes could enhance our understanding of its impact on cancer progression and its potential as a therapeutic target.

### CD47

Cluster of differentiation 47 (CD47), originally named integrin-associated protein, is a transmembrane protein that belongs to the immunoglobulin superfamily and is expressed on a broad range of cell types, including erythrocytes, platelets and lymphohematopoietic cells [211, 212]. A growing body of evidence showed that CD47 is overexpressed in different types of malignancies, such as lung [104], breast [107], bladder [105], hepatic cancers [102] and leukemia [99]. CD47 acts as a ligand for signal regulatory protein α (SIRPα), a protein expressed on phagocytic cells, such as macrophages and DCs [213]. The interaction between CD47 and SIRPα generates a signaling cascade that leads to the inhibition of phagocytosis [212]. Abundant expression of CD47 is utilized by cancer cells as a mean to escape destruction by tumor-associated macrophages [211]. Besides, the CD47-SIRPα signal was found to promote tumor cell proliferation, survival, angiogenesis, metastasis, drug resistance and stemness [211]. Notably, high CD47 expression has been associated with poor prognosis in various solid and hematological malignancies [213]. Given its essential role as a negative ICP, numerous efforts have been made to evaluate the therapeutic potential of targeting the CD47-SIRPα axis in cancer. Antibodies blocking CD47 were shown to reduce tumor size and inhibit metastasis by enhancing cancer cell clearance through macrophages [101, 213]. Additionally, blocking this signal may promote antigen cross-presentation, resulting in T cell priming and activating adaptive anti-tumor immunity [214]. Currently, only ICPs of lymphoid lineage targeting T cell signaling (i.e., PD-1/PD-L1 and CTLA-4) are being used in clinical practice [211]. Therefore, targeting an ICP of myeloid cells and the innate immune system, CD47, serves as a prospective strategy for cancer immunotherapy [101]. To date, clinical trials assessing mAbs targeting CD47 have shown promising results in hematological malignancies, whereas studies on solid tumors are still limited [211].

Numerous studies have demonstrated that CD47 is selectively enriched in the CSC population compared to bulk tumor cells in various malignancies, including AML [99], lung [103, 104], bladder [105], hepatic [102], colorectal [100], pancreatic [106], ovarian [109], esophageal [111] and breast cancer [108]. Consistently, Wang et al. reported that CD47^+^ cells formed significantly larger spheres than CD47^−^ cells. On the other hand, the knockdown of CD47 inhibited stem cell characteristics [99]. Interestingly, blocking CD47 with monoclonal antibodies inhibited tumor growth and effectively eliminated CSCs through both SIRP-dependent [99, 101, 104] and SIRP-independent mechanisms [107].

### CD70

CD70 is a type II transmembrane glycoprotein that belongs to the tumor necrosis factor (TNF) superfamily [215]. It is primarily expressed on antigen-activated immune cells, such as, B cells, conventional T cells, Tregs cells and NK cells [216]. On the other hand, its unique receptor, CD27, is generally expressed on naive T cells, memory B cells, memory T cells, and subsets of NK cells [216]. Under physiological conditions, the activation of the CD70/CD27 axis promotes T cell activation, proliferation and survival as well as enhances B cell differentiation and antibody production [217]. However, CD70 expression is tightly regulated and typically transient, ensuring immune responses, occur only when necessary to maintain immune homeostasis [216, 217]. Conversely, aberrant expression of CD70 has been observed in several types of malignancies, such as RCC and nasopharyngeal carcinoma, leading to lymphocyte apoptosis, T cell exhaustion and immune evasion, which promote tumor progression [216, 218, 219]. Notably, high expression of CD70 in cancer patients has been associated with poor survival [216]. Given its aberrant expression in malignancies, CD70 has become a promising target for cancer immunotherapies, such as blocking antibodies and CAR-T cell therapies [216].

Recent studies have highlighted a link between CD70 and the generation and maintenance of CSCs, demonstrating its critical role in tumor progression, drug resistance and immune evasion. Studies conducted in pancreatic ductal adenocarcinoma (PDAC), AML and GBM revealed that CD70 is differentially upregulated in CSC subpopulations as compared to differentiated tumor cells [131, 136, 137]. Notably, Liu et al. showed that CD70^+^ breast cancer cells, but not CD70^−^ cells, were associated with self-renewal, differentiation potential, and increased tumor sphere formation and lung metastasis [133]. Similarly, in GBM, CD70 expression was found to be essential for sphere formation and proliferation [137]. Moreover, silencing CD70 downregulated stem-related markers (CD44 and SOX-2), inhibited tumor growth and migration, promoted immune suppression by attracting monocyte-derived M2 macrophages, further highlighting its therapeutic potential [134]. Remarkably, CD70-specific CAR-T cell therapy showed profound anti-tumor effects in GBM without observed toxicity [134]. Consistently, blocking the CD70/CD27 axis in AML induced cell differentiation, reduced cell growth and colony formation and improved survival of AML xenograft mice [135].

### CEACAMs

Carcinoembryonic antigen cellular adhesion molecules (CEACAMs) are cell surface adhesion proteins with immunoglobulin-like ectodomains that play significant roles in extracellular and intercellular signaling. They are involved in various complex cellular processes, including cell adhesion, proliferation, angiogenesis, survival, immune cellular regulation, inflammation and tumor suppression [220]. Structurally, CEACAMs consist of a transmembrane region, extracellular immunoglobulin-like domains, and, in some cases, an intracellular domain that mediates signaling. CEACAMs are classified based on their structure into membrane-anchored G protein-coupled receptors (CEACAM5, 6, 7, and 8) or transmembrane proteins (CEACAM1, 3, and 4) [221, 222]. These proteins are expressed in different tissues and cell types like epithelial cells, endothelial cells, vascular cells, immune cells, including neutrophils, macrophages, lymphocytes as well as cancer cells [223]. Their expression is upregulated in response to specific stimuli, such as infection or inflammation [224]. Among the CEACAM family, several members have been repeatedly linked to cancer [221]. Particularly, cumulative evidence from preclinical and clinical data together revealed a more complex influence, in particular for CEACAM1, CEACAM5, and CEACAM6 on cancer and CSCs [221].

CEACAM1, known as CD66a, BGP1 and CC1, is widely expressed across various epithelial cells, on endothelial cells, and within lymphoid and myeloid cells in healthy tissues [221]. In tumor tissues, the expression of CEACAM1 is highly dynamic with contradictory roles being reported. While its downregulation in the early stages of cancer has been linked to disease progression in colorectal and prostate cancer, its overexpression in other types, such as lung and gastric cancer was associated with increased metastasis [221]. Alternative splicing of the CEACAM1 transcript produces multiple isoforms with variations in their extracellular and cytoplasmic domains, altering their capacity for intercellular adhesion and intracellular signaling [221]. While CEACAM1 isoforms with a long (L) cytoplasmic tail were found to suppress T- and B-Cell receptors’ downstream signaling, isoforms with a short (S) cytoplasmic tail were found to modulate the inhibitory effects of CEACAM1-L isoforms, suggesting that the balance between the isoforms enables the tuning of the inhibitory effects of this ICP [225]. The inhibitory effect of CEACAM1 is at least partially mediated by its interaction with the T cell immunoglobulin and mucin domain 3 (TIM3) which results in the inhibition of T cell function and maintaining its tolerance [226]. Moreover, CEACAM1 functions as an inhibitory receptor on NK cells, enhancing cancer cells' ability to avoid immune surveillance [227]. In addition to its role in controlling immune responses, CEACAM1 was found to influence the stemness features of CSCs [112]. For example, CEACAM1, particularly its long isoform (CEACAM1L), plays a crucial role in GBM-initiating cells (GICs) by activating the c-Src/STAT3 signaling pathway, essential for tumorigenesis and CSC maintenance [112]. Notably, the monomeric form of CEACAM1-L cytoplasmic tail induces phosphorylation of c-Src/STAT3 signaling, whereas its oligomerized form inhibits this phosphorylation [112]. Besides, CEACAM^+^ cells from primary cervical tumors exhibit enhanced Notch signaling, metastasis, and stemness features [4]. Significant differences in the invasion, colony formation, and tumor-forming efficiency have also been observed between CEACAM^+^ and CEACAM^−^ cancer cells [113, 228].

CEACAM5 (CEA) functions primarily as an adhesion molecule but has also been implicated in differentiation and immune modulation [229]. Besides, CEACAM5 was found to stimulate cancer progression by promoting cell proliferation and migration [230]. Multiple studies highlighted a link between CEACAM5 and stemness features. For instance, overexpression of CEACAM5 was found to be a reliable marker for CD133^+^ colorectal CSCs [231]. Consistently, a bioinformatics analysis of pancreatic cancer-specific datasets from TCGA and the Cancer Therapeutics Response Portal (CTRP) identified CEACAM5 as a candidate stemness-related inhibitory ICP in pancreatic cancer. The study showed that patients with high infiltration of M1 macrophages and low expression of CEACAM5 had the best overall survival rate [115]. Thus, targeting CEACAM5 with ICP blockers could enhance pancreatic cancer therapy efficacy [115].

Likewise, CEACAM6, also known as CD66c and nonspecific cross-reacting antigen NCA, is a tumor-associated antigen that plays a crucial role in cell adhesion [221, 232]. CEACAM6 overexpression has been detected in various types of cancers and is correlated with a poorer prognosis, making it the most specific tumor marker within the CEACAM family [221, 232]. Moreover, CEACAM6 has recently been identified as a stemness marker in CRC. A study by Gemei et al. showed that colon spheres exhibited high expression of CEACAM6, which was perfectly co-expressed with the CSCs marker, CD133 [117]. Besides, silencing of CEACAM6 in CRC cells inhibited their proliferation and clonogenic potential in vitro*,* as well as tumorigenic potential in vivo [117]*.* These findings demonstrate the potential of CEACAM6 as a therapeutic target to prevent metastasis and eradicate CSCs.

### Galectins

Galectins (Gals) are a family of extracellular β-galactoside-binding lectins that bind to glycoproteins, such as integrins and laminin [119]. Galectins play a key role in regulating various cellular processes, including cell differentiation, adhesion, migration, gene transcription, RNA splicing, cell cycle progression, and apoptosis [233]. Remarkably, alteration in the expression patterns of galectins has been linked to several diseases. Particularly, galectin-1,−3,−7, and −9 production is deregulated during malignant transformation [233]. It has been demonstrated that dysfunction or altered expression of galectins is associated with several characteristics and many cancer hallmarks, such as tumor progression, resistance to apoptotic signals, induction of angiogenesis, cell invasion and metastasis [234]. Besides, the production of galectins by tumor cells is considered one of the key mechanisms for evading immune surveillance, as they are involved in regulating different steps of anti-tumor immune responses [233].

Recently, increasing evidence has begun to shed light on the connection between galectins and cancer stemness [235]. For instance, Tummala et al. demonstrated that the release of galectin-3 (Gal-3) and α-ketoglutarate by malignant hepatocytes promotes the transformation of the hepatic progenitor cells into HCC [119]. While α-Ketoglutarate helps maintain the hepatic progenitor cells in undifferentiated conditions, Gal-3 plays a crucial role in preserving their stemness, expansion and aggressive characteristics [119]. Interestingly, Gal-3 inhibition was found to reduce HCC progression, whereas its expression is linked to poor survival [119]. Moreover, a recent study demonstrated that Gal-3 is implicated in the aggressive nature of uterine serous cancer (USC), promoting cell proliferation, migration, and invasion [120]. Interestingly, the loss of Gal-3 impaired stemness features, as evidenced by reduced colony and sphere formation [120]. On the other hand, the addition of extracellular vesicles containing Gal-3 to Gal-3 knockout cells partially restored their colony formation capabilities [120]. Furthermore, Gal-3 plays a significant role in maintaining ovarian CSCs through the activation of the Notch signaling pathway [121]. Notably, the same study revealed that Gal-3 is highly expressed in advanced stages of ovarian cancer and promotes tumor growth in xenograft models, suggesting that targeting Gal-3 could improve ovarian cancer therapy [121]. In vitro and in vivo studies demonstrated that Gal-3 expression is correlated with tumor stage in triple-negative breast cancer (TNBC) compared to adjacent non-TNBC tissues [123]. A positive correlation among Gal-3, vimentin, and CD44 expression highlights Gal-3's involvement in EMT, maintenance of stem cell characteristics and drug resistance [123]. These findings suggest that targeting Gal-3 may improve prognosis and treatment outcomes in TNBC patients [123]. In addition, a study demonstrated that Gal-3 enhances the formation of lung CSCs by targeting EGFR/c-Myc/SOX-2 axis [122]. Mechanistically, Gal-3 activates EGFR by its carbohydrate-binding activity and stabilizes the c-Myc protein, which binds to the SOX2 gene promoter, thereby enhancing SOX-2 expression [122]. While Gal-3 expression has been associated with CSC characteristics in various cancer types, a study by Ilmer et al. showed that Gal-3^−^ breast CSCs are highly tumorigenic, exhibit a mesenchymal phenotype, and show increased drug resistance with an enrichment of CSC markers (CD24^−^/CD44^+^). In contrast, the study highlighted that Gal-3 expression is associated with an epithelial phenotype (EpCAM^+^ and E-cadherin^+^), reduced drug resistance, and lower tumorigenicity in human breast cancer cells [127].

Similarly, galectin-8 (Gal8) has been implicated in promoting stemness. A study demonstrated that Gal8 enhances autophagy and upregulates stemness markers (CD133, SOX-2, and OLIG2) by activating the mTORC1-TFEB axis [128]. Conversely, inhibition of Gal-8 suppresses tumor growth and prolongs the survival of GBM patients [128]. Moreover, galectin-9 (Gal-9), a ligand of TIM3, strongly promotes the accumulation of β-catenin in AML and enhances self-renewal and propagation of leukemic stem cells (LSCs) upon ligation with TIM3 [129]. Furthermore, a recent study revealed that stem-like cells can differentiate into mature leukemia cells with elevated expression of Gal-9, resulting in immune suppression and the accumulation of dysfunctional CD8^+^T cells [130].

### Regulation of ICP expression in CSCs

The elevated expression of ICPs in CSCs and the TME involves complex intrinsic and extrinsic interactions, although the regulatory mechanisms behind this phenomenon remain incompletely understood. Intrinsic regulation involves molecular pathways or transcription factors within the cancer cells themselves. For instance, PD-L1 expression in cancer cells was reported to be regulated by various intrinsic factors, including mutations in receptors and signaling pathways like PI3K/AKT/mTOR, EGFR, MAPK, PTEN, and IL6/JAK/STAT3 pathways [2, 236–239]. Besides, PD-L1 expression was also found to be induced through activation or overexpression of transcription factors, such as HIF-α, STAT3 and c-Myc [240–242]. Notably, some of these regulators, particularly NOTCH3/mTOR, c-Myc, and HIF-α, have been directly linked to ICP regulation within CSC populations, where their activation correlates with enhanced stemness and elevated PD-L1 expression [76, 77]. While direct evidence linking other pathways to ICP regulation in CSCs is still limited, several studies have independently shown an association between PI3K/AKT, PTEN, EGFR and stemness and ICP (e.g., CD47, PD-L1 and TIM3) regulation in bulk tumor cells [243–245].

For example, TIM-3 expression has been associated with NF-κB and STAT3 signaling in hepatocellular carcinoma, both of which also influence CSC maintenance [246–248]. Similarly, VISTA expression can be regulated through transcriptional networks that are shared with CSC regulatory pathways, including TGF-β/SMAD and HIF1-α [249, 250]. Moreover, many of the pathways that induce the expression of ICP have also been reported to promote EMT, a key hallmark of CSCs, in cancer cells [251, 252]. This overlap suggests a likely role for these pathways in modulating ICP expression in CSCs as well.

On the other hand, the interactions with the TME and immune cells were found to play a critical role in the extrinsic regulation of the ICPs expression. PD-L1 expression can be induced by cytokines such as IFN-γ, TNF-α, IL-10 and IL-4, as well as by other extrinsic stimuli like hypoxia, viruses and chemotherapies [2]. In CSCs, the dysregulation of these processes could coordinate the surface abundance of ICPs and ultimately influence their immune signaling pathways [253–255]. Nevertheless, further research is required to fully elucidate the regulatory mechanisms involved.

## Therapeutic landscape of FDA-approved ICP inhibitors

ICIs are currently used in various solid and hematological malignancies due to their ability to induce durable responses, even in metastatic diseases, offering hope for cancer cure. However, only a limited subset of cancer patients benefits from these therapies. While currently available ICIs are often better tolerated than chemotherapy, they can be associated with autoimmune-related toxicities that might be life-threatening.

Growing evidence indicates that ICPs, such as CTLA-4, PD-1, and PD-L1, play a vital role in modulating both innate and adaptive immune responses. Inhibiting these ICPs can alleviate the constraints on the immune system and enhance the anti-tumor immune response [3, 53]. In the last ten years, the targeting of inhibitory ICP proteins by mAbs has emerged as a crucial component of cancer therapy. Table 3 summarizes currently FDA-approved ICIs.

**Table 3:** Approved immune checkpoint inhibitors (ICIs) for treatment of cancer patients [256]

Class	Drug Name	Year of First FDA approval	Examples of FDA Approved Indications
**CTLA-4 Inhibitors**	Ipilimumab (Yervoy)	2011	Unresectable or metastatic melanoma, adjuvant treatment of melanoma, advanced RCC, MSI-H or dMMR CRC, HCC, metastatic NSCLC, malignant pleural mesothelioma, unresectable advanced or metastatic ESCC
**PD-1 Inhibitors**	Nivolumab (Opdivo)	2014	Unresectable or metastatic melanoma, adjuvant treatment of melanoma, neoadjuvant treatment of resectable NSCLC, metastatic NSCLC, malignant pleural mesothelioma, advanced RCC, cHL, HNSCC, urothelial carcinoma, MSI-H or dMMR CRC, HCC, ESCC
	Pembrolizumab (Keytruda)	2014	Unresectable or metastatic melanoma, NSCLC, malignant pleural mesothelioma, HNSCC, cHL, PMBCL, urothelial cancer, MSI-H or dMMR solid tumors, gastric cancer, esophageal cancer, TNBC
	Cemiplimab-rwlc (Libtayo)	2018	Cutaneous SCC, basal cell carcinoma, NSCLC
**PD-L1 Inhibitors**	Atezolizumab (Tecentriq)	2016	NSCLC, SCLC, HCC, unresectable or metastatic melanoma, alveolar soft part sarcoma
	Avelumab (Bavencio)	2017	Metastatic Merkel cell carcinoma, locally advanced or metastatic urothelial carcinoma, advanced RCC
	Durvalumab (Imfinzi)	2017	NSCLC, SCLC, advanced or metastatic biliary tract cancer, dMMR endometrial cancer, muscle invasive bladder cancer
**LAG-3 inhibitors**	Nivolumab and relatlimab-rmbw (Opdualag)	2022	Unresectable or metastatic melanoma

**CRC**: Colorectal cancer; **cHL**: Classical Hodgkin lymphoma; **dMMR**: Deficient mismatch repair; **ESCC**: Esophageal squamous cell carcinoma; **FDA**: Food and Drug Administration; **HCC**: Hepatocellular carcinoma; **HNSCC**: Head and neck squamous cell carcinoma; **ICIs**: Immune checkpoint inhibitors; **LAG-3**: Lymphocyte activation gene-3; **NSCLC**: Non-small cell lung cancer; **PD-1**: Programmed cell death protein 1; **PD-L1**: Programmed death-ligand 1; **PMBCL**: Primary mediastinal B-cell lymphoma; **RCC**: Renal cell carcinoma; **SCC**: Squamous cell carcinoma; **SCLC**: Small cell lung cancer; **TME**: Tumor microenvironment; **TNBC**: Triple-negative breast cancer

Given that CTLA-4 acts as a co-inhibitory molecule of T lymphocytes, CTLA-4 inhibition is an attractive therapeutic prospect [257]. In 2011, ipilimumab (Yervoy^®^, Bristol-Myers Squibb, USA), a fully human IgG1κ anti-CTLA-4 mAb, became the first ICI to receive FDA approval. Subsequently, ipilimumab received approval for use in different types of malignancies, including unresectable or metastatic melanoma, MSI-H or dMMR metastatic CRC and NSCLC.

Blockade of PD-1/PD-L1 signaling, a mechanism used by tumors to evade T cell immune responses, is another promising cancer immunotherapy strategy [258]. Three anti-PD-1 mAbs, nivolumab, pembrolizumab, and cemiplimab have received FDA approval for the treatment of several cancers [259]. Nivolumab (Opdivo^®^, Bristol-Myers Squibb, USA) is the first fully human immunoglobulin G4 (IgG4) mAb that selectively targets and blocks the interaction between the two ligands PD-L1 and PD-L2 and the PD-1 receptor, thereby restoring cellular immune response [259]. Another PD-1 inhibitor that has gained clinical approval in several cancer types is pembrolizumab (Keytruda®, Merck, USA), a humanized IgG4κ mAb. Pembrolizumab gained approval in 2014 for the treatment of unresectable or metastatic melanoma after its survival benefit was demonstrated in two phase III trials [260, 261]. Interestingly, pembrolizumab is also the first drug to receive universal approval for MSI-H or dMMR-deficient cancer [262, 263]. Cemiplimab (Libtayo^®^, Regeneron Pharmaceuticals, USA) is a third human mAb against PD-1 that was approved by the FDA in 2018 for the treatment of locally advanced or metastatic cutaneous squamous cell carcinoma [264]. Another method of targeting the PD-1/PD-L1 signaling axis is to inhibit PD-L1. There are currently three mAbs approved by the FDA that target PD-L1: atezolizumab, avelumab, and durvalumab.

Relatlimab-rmbw is a LAG-3 inhibitor that is marketed as a fixed-dose combination with nivolumab under the trade name (Opdualag^®^, Bristol-Myers Squibb, USA) and was approved in 2022 for the treatment of previously untreated metastatic or unresectable stage III or IV melanoma [265]. The combination demonstrated superior progression-free survival compared with nivolumab alone in advanced melanoma [265].

Despite the remarkable success of ICIs, a considerable proportion of patients fail to respond to these therapies, highlighting the complexity of the TME and immune system. Besides, while currently available ICIs are often better tolerated than chemotherapy, they can be associated with autoimmune-related toxicities that might be life-threatening. The presence of Tregs, MDSCs, cancer-associated fibroblasts and CSCs within the TME has been implicated in mediating resistance to anti-tumor immunity and ICP-targeted therapies [266]. Particularly, CSCs are well known for their role in driving tumor progression, metastasis and resistance to conventional therapies [5]. Emerging evidence also identifies CSCs as a driver of immunosuppression in multiple types of cancer such as glioma and pancreatic cancer, where they have been positively associated with cold immune phenotype [5, 267].

While a strong positive correlation has been observed between tumor immunogenicity and ICI efficacy, further investigations are necessary to fully understand the underlying determinants of response across various types of cancer [268]. Tumor heterogeneity—both intertumoral and intratumoral—plays a critical role in dampening immune responses and poses a major barrier to effective targeted therapies [269, 270]. Notably, tumor heterogeneity is closely linked to CSCs' plasticity and has been associated with tumor invasion and progression [271]. However, the precise role of CSCs in modulating the responses to ICIs is not fully understood. Besides, it remains unclear whether immunotherapy selectively enriches non-immunogenic CSCs or induces reprogramming of non-CSCs into CSC-like cells [272]. For instance, Gross et al. showed that tumors escaping anti-CTLA4 and anti-PD-1 therapy exhibited an increase in CSC-like populations, with the increase being significantly more pronounced following anti-CTLA-4 treatment compared to anti-PD-1 [272]. This enrichment was driven by endogenous IFN-γ production and was effectively abolished upon administration of IFN-γ–blocking antibodies [272]. This pro-tumorigenic induction of CSCs contrasts with the well-known anti-tumor activities of IFN-γ [273]. Similarly, analysis of single-cell RNA-seq from breast cancer patients pre- and post-pembrolizumab (anti-PD1) treatment showed a significant enrichment in CSC populations, associated with upregulation of stemness-related genes [274]. On the other hand, Wang et al. demonstrated that RCC tumors with high expression of stemness-related genes exhibited increased sensitivity to anti-CTLA-4 therapy, which was accompanied by a suppression of stemness features [65, 161]. Besides, knockdown of PD-L1 was associated with impaired CSC proliferation and downregulation of stemness-related genes. These findings underscore the complexity of the relationship between CSCs and response to ICI therapy, highlighting the need for more comprehensive studies to unravel this dynamic interplay.

## Current advances in ICP targeting in cancer and CSCs

Despite the success of currently approved ICIs, resistance, relapse, and adverse treatment outcomes remain significant challenges. To address these limitations and improve patient outcomes, ongoing research is addressing new therapeutic strategies, including testing novel therapeutic agents, using dual therapy, targeting emerging CSC markers, and devising strategies to manage immune-related adverse events. In this section, current preclinical and clinical trial advances utilizing these strategies are discussed, particularly against emerging ICPs and other immunotherapy targets, including LAG-3, TIM-3, TIGIT, VISTA, B7-H3, indoleamine 2,3-dioxygenase 1 (IDO1), CD47, CD47/SIRPα, OX40 (CD134), glucocorticoid-induced tumor necrosis factor receptor (GITR), inducible T cell co-stimulator (ICOS), CD40, the CD39/CD73/A2AR adenosine pathway, and Siglec-15.

LAG-3 is the third ICP to be clinically validated for cancer therapy; however, its efficacy so far has been only demonstrated in combination with other agents, not as monotherapy [275]. Studies have shown that combination of LAG-3 inhibitors with PD-1/PD-L1 inhibitors possess synergistic activity, leading to stronger activation of T cells [276]. Currently, over 50 LAG-3 targeting agents are being explored in preclinical and clinical studies, aiming to enhance anti-tumor immune responses [277]. The association between LAG-3 and CSCs has not been yet established. Nevertheless, the release of Gal-3, an alternative ligand for LAG-3, by CSCs was found to induce T cell apoptosis and suppress anti-tumor immunity [278]. Besides, co-culture of T cells with CD44^+^/CD90^+^ CSC-like cells in SCLC resulted in upregulation of several ICPs including LAG-3 [50]. Together, these findings imply that LAG-3 blockade, especially in combination with PD-1 or PD-L1, has significant potential for overcoming CSC-mediated immune evasion and enhancing anti-tumor immune responses.

TIM-3 is another key ICP target whose blockade has shown promising therapeutic potential. TIM-3 overexpression was found to be correlated with resistance to PD-1/PD-L1 inhibitors, implicating that it plays a role in treatment failure [279]. Preclinical investigations have demonstrated that inhibiting TIM-3 promotes T cell proliferation and cytokine production [280]. Additionally, dual blockade of TIM-3 and PD-1 has synergistic effects by improving T cell function, suppressing tumor growth, and overcoming anti-PD-1 resistance [281, 282]. Remarkably, TIM-3 has been identified as a key surface marker selectively enriched on LSCs, but not on normal hematopoietic stem cells [283]. Mechanistically, TIM-3 and its ligand Gal-9 promotes activation of canonical Wnt/β-catenin pathway, enhancing CSC self-renewal and maintenance in AML [129, 284]. Kikushige et al. showed that TIM-3^+^ but not TIM-3^−^ AML cells were able to form tumor in vivo, suggesting that TIM-3^+^ subset contains the majority of functional CSC population [285]. Administration of anti-TIM-3 antibodies significantly suppressed AML and eliminated CSCs in preclinical studies [285, 286]. Currently, several TIM-3 inhibitors (e.g., LY3321367, sabatolimab) in combination with other ICIs are being explored for their efficacy and tolerability in clinical trials, and have shown good tolerability and at least partial clinical responses [287–289]. These findings highlight that TIM-3 blockade is a promising strategy for overcoming CSC-mediated resistance mechanisms and enhancing immunotherapy efficacy in both solid and hematologic malignancies.

Immunotherapy strategies targeting B7-H3 are also being explored in preclinical and clinical trials via various approaches, including mAbs, antibody–drug conjugates (ADCs), bispecific and tri-specific antibodies, CAR-T cell and CAR-modified NK therapies, antibody-dependent cellular cytotoxicity (ADCC), and dual-affinity retargeting (DART) technology, which redirects T cells to attack B7-H3-expressing tumors via CD3 [290]. Notably, enoblituzumab, an ADCC-based therapy, has been shown to be effective in prostate cancer by inhibiting B7-H3-mediated immune suppression and promoting cancer cell destruction [291]. In addition to immunotherapy, small-molecule inhibitors targeting B7-H3 are being explored. For example, FDW028, a newly developed FUT8 inhibitor, promoted the lysosomal degradation of B7-H3 through the chaperone-mediated autophagy pathway and demonstrated strong therapeutic efficacy in treating metastatic CRC [292]. Notably, B7-H3 targeted therapies have also demonstrated efficacy against CSC subpopulations in various cancers, including GBM, bone cancer, breast cancer and PCa [86, 293–295]. For instance, Zhang et al. showed that B7-H3 CAR-T cells induced cytotoxicity in both prostate CSCs and bulk of PCa cells, with higher potency against CSCs [86]. Besides, this treatment effectively targeted radio-resistant prostate CSCs, and its combination with fractionated irradiation resulted in enhanced anti-tumor efficacy in PCa and chordoma cancer models [86, 293]. Similarly, knockdown or knockout of B7-H3 using shRNA or B7-H3 gRNAs via the CRISPR/Cas9 dramatically suppressed CD24^low ^CD44^high^ CSC population [84]. Taken together, B7-H3–targeted therapies hold significant promise for enhancing anti-tumor immunity, highlighting the need for additional clinical evaluations.

On the other hand, preclinical studies have demonstrated that TIGIT blockade enhances anti-tumor T cell responses, reduces tumor growth, and restores CD8^+^ T cell potency, particularly when combined with PD-1 or PD-L1 inhibitors [296–298]. Remarkably, tiragolumab, a fully human anti-TIGIT mAb, has shown promising outcomes in multiple clinical studies. In the phase 1b/2 MORPHEUS-Liver trial, adding tiragolumab to atezolizumab and bevacizumab improved the objective response rate (43%) compared with atezolizumab plus bevacizumab alone (11%) in unresectable hepatocellular carcinoma [299]. ​Another phase 2 trial in NSCLC demonstrated that tiragolumab plus atezolizumab achieved a 31.3% ORR versus 16.2% with atezolizumab alone [300]​. Although no studies so far have evaluated the link between CSCs and TIGIT, its ligand CD155 was shown to activate Shh signaling, which is a key stemness pathway. Therefore, dual treatment with TIGIT inhibitors holds great promise for overcoming immunotherapy resistance and improving cancer response rates.

VISTA is another target that has emerged as a critical ICP protein, playing a significant role in suppressing T cell activation and promoting an immunosuppressive TME [301]. Owing to its involvement in tumor progression, several investigational therapies targeting VISTA are under development. For example, HMBD-002, a novel anti-VISTA IgG4 monoclonal antibody, has demonstrated strong preclinical efficacy as monotherapy and in combination with pembrolizumab, significantly inhibiting tumor growth in TNBC and NSCLC models [302]​. In addition, CA-170, an oral small-molecule inhibitor of VISTA and PD-L1, achieved a 75% clinical benefit rate (CBR) and prolonged progression-free survival (PFS) in non-squamous NSCLC patients during a phase II trial [303]​. Emerging strategies include nanoparticle-based approaches [304]. A dual-action lipid nanoparticle (dual-LNP) that co-delivered VISTA-specific siRNA and a TLR9 agonist (CpG) showed superior anti-tumor efficacy, effectively silencing VISTA expression and enhancing cytotoxic T cell responses in melanoma and colon carcinoma models [304]. The use of these novel therapeutic approaches in targeting this emerging pathway shows great promise in overcoming the limitations of current ICIs. Nevertheless, more studies are needed to investigate whether targeting this pathway could overcome CSC-related resistance and relapse.

Indoleamine 2,3-dioxygenase 1 (IDO1) is an enzyme that depletes tryptophan to suppress T cell function, and targeting it represents a potential therapeutic strategy [305]. Notably, IDO1 was found to be highly expressed in GBM and CRC CSCs, where it plays a role in CSC-mediated immune evasion and thus could represent a promising therapeutic approach in cancer immunotherapy beyond traditional ICIs or CAR-T cell therapies [172, 306]. While surface checkpoint receptors can be targeted with antibody-based therapies, IDO1 operates intracellularly, making small-molecule drugs the most effective approach. Therefore, a growing number of small-molecule drugs are currently in preclinical and clinical development as IDO1 inhibitors [307]. Among these, indoximod is the most extensively studied, having received orphan drug designation from the U.S. FDA for treating stage IIb to stage IV melanoma [307]. It has been shown that IDO1 is closely interconnected with CTLA-4 and the PD-1/PD-L1 pathways, contributing to CSC resistance against checkpoint inhibitors [308, 309]. In particular, combining IDO1 inhibitors with PD-1 or CTLA-4 blockade has demonstrated enhanced anti-tumor effects in early phase clinical trials, particularly in melanoma and advanced GBM models [308, 309]. However, subsequent larger studies have yielded mixed outcomes. For instance, ECHO-301/KEYNOTE-252 phase 3 trial showed that combining IDO1 inhibitor (Epacadostat) with pembrolizumab did not enhance progression-free survival or overall survival compared to pembrolizumab alone in melanoma patients [310]. Besides, clinical trials combining IDO1 inhibitors with CTLA-4 blockade in melanoma patients reported toxicities, leading to the discontinuation of some studies [311]. While IDO1 inhibitors may hold promise in overcoming immunotherapy resistance and improving patient outcomes, their current effectiveness remains uncertain, highlighting the need for further investigation and careful patient selection in combination strategies.

Additionally, the CD47/SIRPα axis is being actively explored for its role in various cancers, especially hematological malignancies, to restore macrophage-mediated phagocytosis of tumor cells [312–314]. It has been shown that inhibition of CD47 with monoclonal antibodies suppressed tumor growth and effectively eliminated CSCs across various malignancies [99, 101, 104, 107]. Liu et al. reported that anti-CD47 effectively suppressed tumor growth and prolonged survival in mouse xenograft model engrafted with lung cancer and CSCs [104]. Similarly, anti-CD47 antibody enabled phagocytosis of AML CSCs and inhibited their growth in vivo [99]. Remarkably, several agents that target CD47 pathway are showing promise in clinical trials. Magrolimab, a mAb anti-CD47 antibody, has demonstrated efficacy in combination with rituximab in patients with relapsed/refractory indolent non-Hodgkin lymphoma, achieving a 52% ORR with a 30% complete response rate and a median duration of response (DOR) of 15.9 months [315]. Furthermore, the anti-CD47 antibody CC-90002 was investigated in a phase 1 trial for AML and high-risk MDS, which demonstrated macrophage-mediated phagocytosis in preclinical models but limited clinical efficacy, leading to trial discontinuation [316]​. In addition, the bispecific fusion protein SL-172154, which combines CD47 inhibition with CD40 agonism, showed promising immune activation in a phase 1 trial for platinum-resistant ovarian cancer, with 22% of patients achieving stable disease and significant increases in CD8^+^ T cell infiltration [317].

Other emerging ICPs currently being explored for therapeutic targeting include OX40 (CD134), GITR, ICOS, CD40, the CD39/CD73/A2AR adenosine pathway, and Siglec-15. In particular, agonists of the costimulatory pathways OX40 (CD134), GITR, and ICOS have demonstrated encouraging outcomes in preclinical and early-phase trials [318]. On the other hand, advancements in antibody engineering and mechanistic insights are currently driving the development of next-generation Fc-engineered and multi-specific CD40 agonists designed to enhance potency while minimizing toxicity, especially in combination therapies [319]. The immunosuppressive CD39/CD73/A2AR adenosine pathway, known for impairing T cell and NK cell functions, is also gaining attention as a potential therapeutic target, as adenosine generated within the TME significantly suppresses immune responses [320]. Finally, sialic acid-binding immunoglobulin-type lectin 15 (Siglec-15), an emerging immune checkpoint protein, has been investigated as a novel checkpoint to counteract CSC-induced immune suppression [321–323].

## Integrating CSC-targeted therapies with immune checkpoint blockade to counteract therapeutic resistance

To date, it remains unclear whether ICI therapy alone is sufficient to eliminate CSCs. Given the importance of CSCs in suppressing anti-tumor immunity, resisting conventional therapies, and promoting tumor relapse, targeting key regulators of CSCs may offer new therapeutic opportunities. Particularly, combining ICIs with therapies targeting CSCs or their regulatory signaling pathways holds significant promise for achieving more durable and sustained anti-tumor responses as well as overcoming resistance to ICIs. In the following section, we highlight suggested combination strategies that may improve CSC sensitivity to ICP-based treatments and the molecular mechanisms involved (Table 4).

**Table 4:** Overview of CSC-directed and ICIs based combination therapies

Cancer type	ICI	Add-on therapy	Effect on ICI therapy response	Ref
HNSCC	Anti-PD-1	BMI1 inhibition	Enhanced CD8^+^ T cells recruitment and activation, and elimination of BMI1^+^ CSCs	[60]
Melanoma	Anti-PD-L1 and anti-CTLA-4	ALDH^high^ CSC lysate-pulsed dendritic cell (DC) vaccine	Eliminated ALDH^high^ CSCs and promoted anti-tumor immune responses	[324]
Squamous cell carcinoma (SCC)	Anti-CTLA-4	TGF-β inhibitors	Following CTLA-4 or TGF-β blockade, or C80 deletion, CSCs become vulnerable, thereby reducing the likelihood of tumor relapse after adoptive cell transfer (ACT) therapy	[325]
Merkel cell carcinoma (MCC)	PD-1/PD-L1 blockade	Panobinostat (HDACi)	Upregulated HLA class I surface expression, and increased CD8^+^ T-cell infiltration. However, additional studies are needed to validate these findings and investigate clinical relevance	[326]
Unresectable stage III/IV melanoma	Anti-CTLA (ipilimumab)	Guadecitabine (DNA hypomethylating agent)	26% objective response rate, accompanied by upregulation of HLA class I on tumor cells, an increase in CD8+, PD-1^+^ T cells and in CD20^+^ B cells	[327]
NSCLC	-	Bispecific antibody targeting c-MET and CTLA-4	Inhibited CD166^+^ CSCs and tumor development, suppressed Tregs and upregulated effector T cells	[71]
Cholangiocarcinoma	Anti-PD-1	Verteporfin (YAP inhibitor)	Inhibited CSCs and reduced tumor burden	[328]
GBM	Anti-PD-1 and anti-CTLA-4	Oncolytic herpes simplex virus expressing IL-12	Cured CSC-derived GBM	[329]
RCC	Anti-PD-1	DCLK1-IN-1	Enhanced immune-mediated cytotoxicity and downregulated stemness and EMT markers	[181]
TNBC	Anti-PD-L1	LCOR mRNA therapy with extracellular vesicles (EVs)	Promoted ICIs efficacy by boosting tumor APM signaling	[330]
CRC	Anti-PD-L1	Mithramycin A	Suppressed tumor growth, promoted CD8^+^ T cell infiltration, reduced MDSCs and anti-inflammatory macrophages	[331]
Melanoma and CRC	Anti-PD-1	Metformin	Improved anti-tumor activity and enhanced T cell activity	[332]

B-lymphoma Mo-MLV insertion region 1 (BMI1) is a key component of polycomb repressive complex 1, which plays a critical role in regulating stemness and immune evasion [333]. Notably, treatment of HNSCC with cisplatin plus anti-PD-1 induced enrichment of BMI1^+^ CSCs. Interestingly, the inhibition of BMI1 effectively suppressed the self-renewal capacity of CSCs and enhanced their depletion, leading to the suppression of tumor progression [60]. Besides, BMI1 blockade enhanced CD8^+^ T cell activation and recruitment by promoting IRF3-mediated transcription and suppressing H2A ubiquitination [60]. Similarly, the addition of BMI1 inhibitor to anti-PD-L1 immunotherapy following pre-chemoradiation treatment resulted in synergistic anti-tumor activity in a lung cancer mouse model [334]. These findings highlight BMI1 inhibition as a promising approach to boost the efficacy of anti–PD-1/anti-PD-L1 therapy and inhibit tumor relapse.

Dendritic cell (DC)-based vaccines utilize DCs' abilities to elicit robust immune responses by linking innate and adaptive immunity [335]. Over the past decade, several clinical trials demonstrated that DC-based vaccines loaded with tumor antigens could effectively induce specific anti-tumor T cell responses in different types of tumors and achieved long-term clinical benefits in a subset of patients [335, 336]. Remarkably, DCs loaded with CSC lysates have also been shown to induce potent anti-tumor immune responses in various malignancies [336]. For instance, a DC vaccine loaded with Nanog peptide, a key stemness regulator, elicited a robust anti-tumor response against CSCs in ovarian cancer [337]. Besides, DCs pulsed with ALDH^high^ CSC lysates effectively suppressed tumor relapse and prolonged survival [338]. Therefore, CSC-targeted DC-based vaccines are currently being evaluated in multiple clinical trials (e.g., NCT02178670, NCT03548571, NCT00846456) [336, 339]. Moreover, the combination of ICIs with CSC-DC-based vaccines has been found to synergistically reduce tumor growth. Particularly, the triple combination of anti-PD-L1, anti-CTLA-4 and CSC-DC vaccine significantly eliminated ALDH^high^ CSCs, which was accompanied by improved T cell expansion, suppressed TGF-β release, increased IFN-γ, and promoted CD8^+^ T cell response against melanoma CSCs [324]. Similarly, administration of an ALDH^high^ CSC-DC vaccine along with anti-PD-L1 significantly prolonged survival and inhibited tumor recurrence in a murine model of squamous cell carcinoma [338]. Taken together, these findings suggest that combining DC vaccines with ICI therapy might be a promising strategy to enhance the therapeutic outcomes and prevent tumor recurrence.

Emerging evidence indicates that TGF-β contributes to resistance to ICIs and has even been associated with hyperprogressive tumors upon treatment with anti-PD-1 therapy [340]. Mechanistically, the release of TGF-β has been linked to increased PD-L1 expression, thereby facilitating immune evasion [341, 342]. Therefore, combining TGF-β inhibitors with anti-PD-1 or anti-PD-L1 has been proposed as a strategy to augment anti-tumor immune responses and enhance tumor eradication [343, 344]. Simultaneous targeting of TGF-β and PD-L1 through bifunctional antibodies is currently being evaluated in clinical trials [345, 346]. Notably, several studies showed that TGF-β plays an important role in the maintenance and expansion of CSC populations across various malignancies, including GBM, breast cancer and skin cancer [347]. Interestingly, TGF-β inhibition effectively targeted CD44^high^/ID1^high^ CSCs isolated from GBM patients [348]. Similarly, blocking TGF-β signaling in CD80^+^ CSCs from SCC enhanced their sensitivity to adoptive cytotoxic T cell transfer-based immunotherapies [325]. These findings suggest that the enhanced anti-tumor immune responses observed with combined ICI therapy and TGF-β blockade may at least be partially mediated through enhanced targeting and eradication of CSCs.

One of the mechanisms contributing to resistance against ICIs is the downregulation of MHC molecules [349]. Several studies have reported suboptimal expression of HLA class I molecules and downregulation of their downstream antigen-presenting machinery signaling in CSCs across different types of tumors [350–352]. This impaired antigen presentation reduces tumor immunogenicity and limits T-cell-mediated recognition. Interestingly, restoring HLA expression was accompanied by enhanced sensitivity to anti-PD-1 and anti-PD-L1 therapies [353, 354]. These findings suggest that combining MHC-upregulating agents, such as DNA methyltransferase inhibitors and histone deacetylase inhibitors (HDACi), with ICIs may improve therapeutic responses by enhancing tumor visibility to the immune system [355, 356].

Cellular mesenchymal-to-epithelial transition factor (c-MET) has been implicated in promoting tumor progression, metastasis and angiogenesis, whereas its inhibition has shown survival benefits in a phase II clinical trial [71, 357]. Li et al. developed a bispecific antibody, that simultaneously targets c-MET and CTLA-4, which effectively target CD166^+^ lung CSCs, and inhibit tumor development, proliferation, and migration [71]. The observed anti-tumor effects were associated with suppression of Tregs and upregulation of effector T cells.

Verteporfin, a YAP1/TAZ signaling inhibitor, has demonstrated in vivo anti-tumor efficacy along with the ability to target CSCs [358]. The combination of anti-PD-1 and verteporfin was found to augment the anti-tumor effects in cholangiocarcinoma as compared to either agent alone [328].

Oncolytic viruses (OVs) represent a novel class of anticancer agents that selectively replicate within and kill cancer cells while taking advantage of the virus-induced inflammation and host immune responses to OV which drives cancer cell death and promotes anti-tumor immunity [359]. GBM, one of the most immunosuppressive ‘cold’ tumors, responds poorly to ICIs and is known to be enriched with CSCs. In an in vivo immunocompetent CSC-derived GBM model, Saha et al. revealed that a triple combination of oncolytic herpes simplex virus expressing IL-12, anti-PD-1 and anti-CTLA-4 effectively cured tumor, whereas neither single nor dual combination was effective in overcoming the immune suppression in GBM [329].

DCLK1-IN-1 is a novel small-molecule kinase inhibitor targeting Doublecortin-like kinase 1 (DCLK1) that was found to inhibit stemness and EMT signaling in RCC [181]. Interestingly, DCLK1-IN-1 elicited a strong anti-tumor immune response, which was further augmented upon combination with anti-PD-1 therapy [181].

Ligand-dependent corepressor (LCOR) acts as a differentiation factor in normal and malignant breast stem cells. Notably, LCOR was identified as a key activator of the antigen presentation machinery, and studies have shown that ICIs selectively enrich LCOR^low^ CSCs, suggesting that it plays a role in regulating the response to ICIs [330]. Pérez-Núñez et al. showed that combining extracellular vesicle LCOR-mRNA therapy with anti-PD-L1 suppressed metastasis and overcame therapeutic resistance in TNBC preclinical models [330].

Mithramycin A (Mit-A), a polyketide antibiotic, has been identified as a CSC targeting agent with the ability to kill CSCs in vitro and in vivo [360]. Dutta et al. evaluated its combination with anti-PD-L1 therapy and interestingly found that the combination significantly reduced tumor growth, which was associated with enhanced CD8^+^ T cell infiltration, decreased MDSC and anti-inflammatory macrophages, transforming the TME from being immunologically ‘cold’ to ‘hot’ [331].

Metabolic dysregulation, including alterations in oxidative energetics, has been suggested as a barrier to effective anti-tumor immunity by contributing to the generation of a hypoxic TME [332]. The rate at which cancer cells consume oxygen has been correlated with their sensitivity to ICI therapy. Scharping et al. showed that metformin, an oral anti-diabetic drug, inhibits oxygen consumption in cancer cells, reducing the intratumoral hypoxia. Besides, metformin has also been shown to directly target CSCs [361]. While metformin alone exhibited weak anti-tumor activity in aggressive tumors, its combination with anti-PD-1 therapy led to enhanced T cell activity and tumor eradication [332]. These findings suggest that remodeling the hypoxic TME might be a promising strategy for enhancing the sensitivity to ICIs.

## Bridging the gaps: future directions in CSC-targeting strategies

Despite this promising progress in targeting CSCs and modulating the immunosuppressive TME, significant gaps remain that need to be addressed to effectively eradicate CSCs. Although the expression of ICPs is correlated with aggressive tumor behavior and stemness, the full spectrum of ICP receptors expressed by CSCs is still not fully characterized. Additionally, definitive evidence supporting the use of specific ICPs as biomarkers or therapeutic targets for CSC populations is lacking; therefore, further studies in this area are needed. Current clinical trials predominantly focus on targeting bulk tumor cells, with limited investigations into CSC-specific markers and their contributions to therapeutic resistance. These limitations may stem from the inherent heterogeneity of CSCs, in which many of their characteristics remain poorly characterized across various types of tumors. While numerous stemness markers have been identified, the majority lack sufficient specificity and generalizability across various CSC populations.

The impact of CSCs on immune evasion and ICP regulation also remains underexplored. A deeper understanding of the interplay between CSCs and ICPs could inform the development of more precise and durable therapeutic strategies. Although dual blockade approaches, such as LAG-3/PD-1 (e.g., relatlimab and nivolumab) and CD47/SIRPα (e.g., magrolimab and rituximab) combinations, have shown clinical efficacy, the mechanism of action of dual treatment options in overcoming resistance within the CSC niche remains poorly understood. Additionally, emerging ICP targets, such as VISTA, Siglec-15, and adenosine pathway modulators (CD73/A2AR), which are implicated in immune suppression and CSC survival, remain underexplored, with limited translational data from preclinical models to clinical trials. Similarly, costimulatory agonists such as OX40, GITR, and ICOS, which enhance T cell activation, have demonstrated potential in preclinical and clinical studies, but their efficacy against CSCs remains inconclusive due to a lack of thorough understanding of the signaling pathways involved.

A major limitation in current CSC research lies in the widespread use of immunodeficient mouse models lacking a functional adaptive immune system, which may not always reflect the actual complexities of tumor immunobiology. Similarly, many in vitro studies assess CSCs in isolation from their TME, further limiting translational relevance. To overcome these gaps, experimental protocols for studying CSCs should be optimized and validated in immunocompetent models that enable contact with the immune system. The adoption of three-dimensional (3D) coculture system, such as patient-derived organoids, offers a more physiologically relevant platform to study CSC interactions with immune effectors and the surrounding microenvironment. Combined with advances in single-cell and spatial transcriptomic technologies, these models can provide high-resolution insights into CSC–immune crosstalk and reveal novel therapeutic targets.

Moreover, the absence of comprehensive genomic, epigenetic, and immunological characterization of ICPs in CSCs hinders the identification of new target molecules for rational drug design. Furthermore, since many signaling pathways that regulate CSCs are shared with normal stem cells, not all identified CSC-associated regulatory factors are suitable therapeutic targets.

Further research is also warranted to develop novel strategies for mitigating immune-related adverse events associated with various available ICIs. A key concern is that targeting cancer cells expressing ICPs may deplete immune cells that also express these molecules, causing toxicities. One suggested solution to overcome this challenge is to utilize bispecific antibodies designed to selectively target cells that co-express ICPs with additional tumor-specific antigen, thereby enhancing therapeutic precision while minimizing off-target immune adverse events.

Apart from targeting ICPs, the development of newer anti-tumor agents and treatment strategies should tackle the various mechanisms of interaction between the CSCs and their TME. Emerging immunotherapeutic approaches, such as CSC-specific CAR T cells, DC vaccines loaded with stemness-associated antigens, and adoptive transfer of tumor-reactive T cells hold promise in targeting CSCs more effectively. The integration of these strategies with current immunotherapies may ultimately enhance treatment durability, reduce relapse rates, and improve long-term patient outcomes.

## Conclusion

It has become increasingly accepted that the presence of a rare subpopulation of cells within tumors endowed with self-renewal capacity and stemness properties, denominated as CSCs, is responsible for tumor metastasis, recurrence and resistance to therapy [7, 46]. Moreover, these cells were found to display immunomodulatory functions and thus escape immune recognition. Mechanisms behind CSC immunological characteristics involve suboptimal expression of HLA molecules, secretion of inflammatory cytokines and differential expression of inhibitory ICPs [45, 46]. The latter mechanism gained immense interest over the past few years, especially upon approval of new ICIs and expansion of their clinical applications. CSCs from different tumors were found to differentially express several inhibitory ICPs, such as PD-L1/PD-1, CTLA-4, B7-H3, B7-H4, CD155, CD200, VISTA, TIGIT, CD47, CD70-CD27, CEACAMs and galectins. This dysregulation was associated with higher immune evasion, stemness features, and more aggressive tumor behavior, including higher proliferation, invasion, metastasis and worse clinical prognosis.

The fact that only a subset of patients responds to available ICIs, mainly targeting PD-1/PD-L1 and CTLA-4 axes, highlights the importance of the other ICPs in mediating tumor cells' immune evasion. These checkpoints may mediate immune resistance through a distinct or complementary pathway to PD-1/PD-L1 and CTLA-4. Although available studies showed a correlation between the expression of some ICPs and aggressive tumor behavior, including stemness, the full array of ICP receptors expressed by CSCs has not been fully investigated. Besides, definitive evidence supporting the role of ICPs as potential biomarkers or as a therapeutic approach for targeting the CSC population remains lacking. Therefore, more studies are needed to identify and explore the effects of targeting ICPs that are differentially expressed in CSCs. The gain of the genomic, epigenetic and immunological characterization of ICPs in CSCs will contribute to the identification of new target molecules to be exploited for the rational design and optimization of new targeted immunotherapeutic interventions; or as biomarkers for predicting clinical outcome and therapeutic responses of cancer patients.

## Data Availability

No datasets were generated or analysed during the current study.

## Abbreviations

ALDH: Aldehyde dehydrogenase
AML: Acute myeloid leukemia
B7-H3: B7 homolog 3
B7-H4: B7 homolog 4
CD: Cluster of Differentiation
CD47: Integrin-associated protein
CD70: Tumor necrosis factor ligand superfamily member 7
CD155: Poliovirus receptor
CD200: OX-2 membrane glycoprotein
CEACAMs: Carcinoembryonic antigen-related cell adhesion molecules
CICs: Cancer-initiating cells
CRC: Colorectal cancer
CSCs: Cancer stem cells
CSF: Cerebrospinal fluid
CTLA-4: Cytotoxic T-lymphocyte-associated protein 4
EMT: Epithelial–mesenchymal transition
ESCC: Esophageal squamous cell carcinoma
FGF-2: Fibroblast growth factor 2
FOXD3: Forkhead box D3
GBM: Glioblastoma
HCC: Hepatocellular carcinoma
HER2: Human epidermal growth factor receptor 2
HNSCC: Head and neck squamous cell carcinoma
HPV: Human papillomavirus
ICGC: International Cancer Genome Consortium
ICPs: Immune checkpoint proteins
IL: Interleukin
JAK/STAT: Janus kinase/signal transducers and activators of transcription
Ly6E/K: Lymphocyte antigen 6 complex, locus E/K
LSCs: Leukemia stem cells
mAb: Monoclonal antibody
mRNAsi: mRNA stemness index
mTOR: Mechanistic target of rapamycin signaling
MMPs: Matrix metalloproteinases
NF-κB: Nuclear factor kappa-light-chain-enhancer of activated B cells
NSCLC: Non-small cell lung cancer
PD-1: Programmed cell death protein 1
PD-L1: Programmed death-ligand 1
PDX: Patient-derived xenograft
PI3K/AKT: Phosphoinositide 3-kinase/Protein kinase B pathway
RCC: Renal cell carcinoma
Sca-1: Stem cell antigen-1
SHP-1/2: Src homology 2-containing protein tyrosine phosphatases-1/2
TCGA: The Cancer Genome Atlas
TIGIT: T cell immunoreceptor with Ig and ITIM domains
VISTA: V-domain immunoglobulin suppressor of T cell activation
